# Relationships between the Intakes of Human Milk Components and Body Composition of Breastfed Infants: A Systematic Review

**DOI:** 10.3390/nu15102370

**Published:** 2023-05-18

**Authors:** Isabella Norrish, Azhar Sindi, Vanessa S. Sakalidis, Ching Tat Lai, Jacki L. McEachran, Mya Thway Tint, Sharon L. Perrella, Mark P. Nicol, Zoya Gridneva, Donna T. Geddes

**Affiliations:** 1 School of Molecular Sciences, The University of Western Australia, Crawley, WA 6009, Australia; isabella.norrish@icloud.com (I.N.); vanessasakalidis@gmail.com (V.S.S.); ching-tat.lai@uwa.edu.au (C.T.L.); jacki.mceachran@uwa.edu.au (J.L.M.); sharon.perrella@uwa.edu.au (S.L.P.); donna.geddes@uwa.edu.au (D.T.G.); 2 Division of Obstetrics and Gynaecology, The University of Western Australia, Crawley, WA 6009, Australia; azhar.sindi@research.uwa.edu.au; 3 College of Applied Medical Sciences, Umm Al-Qura University, Makkah 24381-8156, Saudi Arabia; 4 Singapore Institute for Clinical Sciences (SICS), Agency for Science, Technology and Research (A * STAR), Singapore 117609, Singapore; mya_thway_tint@sics.a-star.edu.sg; 5 Human Potential Translational Research Programme, Yong Loo Lin School of Medicine, National University of Singapore, Singapore 117597, Singapore; 6 School of Biomedical Sciences, The University of Western Australia, Crawley, WA 6009, Australia; mark.nicol@uwa.edu.au

**Keywords:** systematic review, human milk intake, early life nutrition, breastfeeding, lactation, dose, growth, infant body composition, macronutrients, bioactive molecules

## Abstract

Human milk provides all of the elements necessary for infant growth and development. Previous studies have reported associations between breastfeeding and a reduced risk of developing obesity and late-onset metabolic disorders; however, the underlying mechanisms are poorly understood. Recently, intakes of human milk components have been associated with infant body composition, which is likely partially implicated in the reduced risk of developing childhood obesity among breastfed infants. In this systematic review, we searched electronic bibliographic databases for studies that explored relationships between the 24 h intakes of human milk macronutrients and bioactive components and infant body composition and/or growth parameters. Of 13 eligible studies, 10 assessed relationships of infant body composition and growth outcomes with human milk macronutrients, while 8 studies assessed relationships with human milk bioactive components. Significant time-dependent relationships with infant anthropometrics and body composition were found for intakes and no relationships for concentrations of several human milk components, such as lactose, total protein, and human milk oligosaccharides, suggesting that measuring concentrations of human milk components without quantifying the intake by the infant may provide a limited understanding. Future studies investigating the effect of human milk components on infant growth and body composition outcomes should consider measuring the actual intake of components and employ standardised methods for measuring milk intake.

## 1. Introduction

Human milk (HM) is a biodynamic matrix of individual components shaped by thousands of years of evolution to provide optimal nutrition for infant protection, development, and health [1,2,3]. This multifunctional substance is composed of nutrients and bioactive factors that provide not only nutrition but developmental and appetite-regulating cues, as well as protection against infectious and non-communicable diseases (NCDs) [4]. Obesity remains a significant concern to the global population and NCDs linked to obesity, such as cardiovascular disease and diabetes, are the leading causes of mortality, and are continuing to rise in prevalence worldwide [5,6]. HM is species-specific and remains unmatched by alternative diets, with a dose–response effect observed for cognition and other health benefits across the lifespan [7,8]. The protective effect of HM has been quantified as a function of exclusivity and duration, with longer breastfeeding exclusivity and duration of breastfeeding incurring a greater reduction in the risk of developing infectious and chronic diseases in both the mother and the infant [9,10]. Moreover, the lifelong risk of NCDs could be modifiable through the early programming effects of nutrition and body composition (BC) development on later obesity [11,12].

While the literature suggests that HM is protective against the development of overweight and obesity in a dose-responsive manner [13,14,15], the underlying mechanisms are yet to be elucidated. Both rapid weight gain in infancy [16] and the elevated protein content in formula [17,18] are associated with an increased risk of obesity later in life. Therefore, clarifying the interplay between the intake of HM and its specific components, including bioactive factors and macronutrients, is critical in our understanding of early programming, infant metabolism, appetite regulation, and subsequent infant growth and BC among breastfed infants.

HM composition is largely conserved across populations and remains relatively stable after the transition from colostrum to mature milk [19]. Nonetheless, it varies throughout the day and throughout lactation [20]. Each breastfeeding dyad is unique, displaying individualised breastfeeding behaviour, including a wide variation in both breastfeeding frequency and volume of milk consumed [21,22,23,24]. Breastfed infants self-regulate their milk intake (MI) in a supply–demand fashion according to their individual needs. Twenty-four-hour MI volumes are highest and most stable between 1 and 6 months of age [25,26], with breastfed infants taking an average of 788 ± 169 g of HM (range: 478–1356 g) over a 24 h period and 101.4 ± 15.6 g per breastfeed [22]. Twenty-four-hour MI volumes also remain substantial in later lactation [27] and are higher in healthy populations [28].

Previous research has shown a positive relationship between the 24 h MI and infant growth [27,29,30]. In a cohort study of 4-month-old exclusively breastfed infants, 24 h MI accounted for approximately 28% of the variability in the rates of infant weight gain [21]. These results prompt the question as to how the intakes of multiple components of HM, working either in isolation or synergistically, contribute to infant growth and BC development.

Studies often demonstrate conflicting relationships between the concentrations of HM components and infant BC and growth. Inconsistent results may be explained, in-part, by the heterogeneity in methods and study designs. As there is large variation in MI, and thus HM component intakes, between infants, relying on the concentration of HM components alone may be misleading. This systematic review thus aimed to explore the relationships between 24 h intake of HM macronutrients and bioactive molecules and infant BC and growth and summarise the key HM factors that may be implicated in the development of BC and growth in breastfed children.

## 2. Materials and Methods

### 2.1. Search Strategy

This systematic review followed the Preferred Reporting Items for Systematic Reviews and Meta-Analyses (PRISMA) procedure for identification, screening, and eligibility of the studies [31]. An extensive search was performed in the electronic databases MEDLINE, EMBASE, and CINAHL, on 17 May 2022, using MESH and free text terms, adapted to each database covering the timeframe from 1937 to 17 May 2022. The search strategies are provided in the Supplementary Material (Tables S1–S4). After article screening, the references of selected full texts were cross-checked to identify additional eligible papers for inclusion. This review focused on studies that investigated the relationships of the 24 h intake of HM components, including macronutrients and bioactive components, such as metabolic hormones and immunomodulatory proteins, with infant BC and growth outcomes of infants/children aged 0–2 years.

### 2.2. Eligibility Criteria and Selection of Articles

Studies were included if they met the following criteria: (a) original studies; (b) focused on healthy term breastfed human infants and children aged between 0 and 2 years; and (c) investigated the relationship between the 24 h intake of HM components and infant BC and/or growth parameters. Studies were excluded if they (a) used animal models; (b) investigated infant formula or donor milk only; (c) measured intakes of contaminants in HM, such as heavy metals and organic pollutants; (d) measured 24 h MI but focused only on the relationships of concentrations of HM components without measuring/analysing intake or dose by the infant; (e) measured 24 h MI but focused only on the relationships of 24 h MI with infant BC/growth; (f) estimated/predicted but did not measure 24 h MI; (g) only measured/analysed energy intake; (h) only conducted between group comparison (e.g., infants of mothers with obesity and infants of mothers with normal weight, with the exception of infant BC outcome grouped as low/medium/high); or (i) mothers or infants were not considered healthy (e.g., preterm infants, growth-stunted/malnourished infants/children, mothers with malnutrition or mastitis). Review papers, conference abstracts, case reports/series, editorials and letters to the editor, and papers written in languages other than English were also excluded.

### 2.3. Data Extraction

Returned titles and abstracts were exported into the review software Rayyan [32]. After duplicate removal, the remaining items were screened by two researchers (Z.G. and V.S.) for inclusion based on their titles/abstracts. Eligible studies for inclusion were retained to the screen as full texts. Z.G. and V.S. screened all full texts independently for inclusion. Disagreements between the researchers were resolved by discussion or by involving an additional reviewer (D.T.G.).

For each selected full text, data were extracted and summarised by (a) component type: macronutrients or bioactive components; (b) study type, sample size, and infant age; (c) methods: HM sample type and timing of sampling, assays/analysis of HM components, 24 h MI, and anthropometry/BC measurements; and (d) results: 24 h MI, component concentration and 24 h intake, and statistical associations/correlations between the intake of components (and concentration if available) and infant growth and BC measurements. As differences in study design, methodology, and BC/growth outcomes assessed do not allow for an appropriate meta-analysis of these limited data, diagrammatic data summaries were created to assist the understanding of heterogeneous results.

### 2.4. Quality Assessment

Two reviewers (V.S. and A.S.) conducted an independent quality assessment of the selected articles using the National Institute for Clinical Excellence (NICE) methodological checklist for cohort studies [33]. Studies were rated on the risk of selection, performance, and attrition and detection bias. Studies were given an overall rating of low, high, or unclear risk of bias and the final score. The scores were agreed upon by consensus or by involving an additional reviewer (Z.G.).

## 3. Results

### 3.1. Synthesis

**Figure 1:**
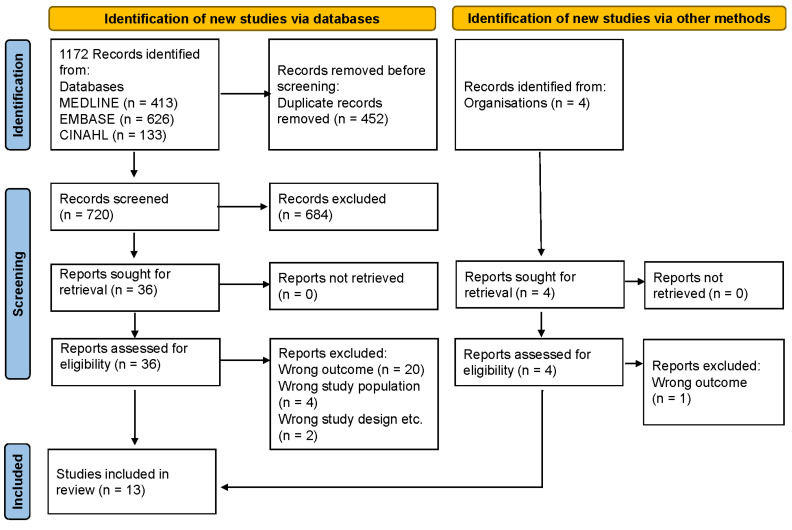
PRISMA diagram.

A total of 1172 titles were identified and, after duplicate removal, 720 abstracts were screened for eligibility (Figure 1). Of these, 36 met the inclusion criteria and were eligible for full-text assessment. Additionally, 4 studies were identified through reference searching/organisations and were also eligible for full-text assessment. After full-text screening, a total of 13 studies were included in the review [29,34,35,36,37,38,39,40,41,42,43,44,45]. Twenty-one studies were excluded for measuring different outcomes (e.g., measuring 24 h MI but analysing/reporting only the results of concentration rather than intake of components), of which 3 studies were excluded as they approximated rather than measured 24 h MI, conducting a test-weighing (with infants fasting for at least 2 h prior to the procedure) before and after a single breastfeed, combined with an average reported breastfeeding frequency, bottle weighing of expressed milk, and/or 3-day food intake [46,47,48]. Four studies were excluded for assessing an unhealthy study population, focusing on malnourished rather than healthy dyads, and two studies were excluded for an inappropriate study design, such as a secondary review of data.

Of the 13 observational studies conducted on seven cohorts and published between 1993 and 2022, 5 assessed relationships of infant BC and growth with intakes of HM macronutrients [29,34,39,42,45] (Table A1), 3 assessed relationships with intakes of HM bioactive molecules [36,40,43] (Table A2), and the remaining 5 studies measured a combination of both macronutrients and bioactive components [35,37,38,41,44]. One cohort study [49] published six separate papers on intakes of HM components [35,38,40,41,42,43] and another cohort study produced two papers [36,37].

### 3.2. Participant Charcteristics and Stage of Lactation

Study populations included breastfeeding dyads from the United Kingdom [34], Russian Federation [44], USA [45], and Australia [29,35,36,37,38,39,40,41,42,43]. Sample sizes ranged from 11 to 174 participants and between 154 and 732 HM samples. HM was collected at two stages of lactation: transitional (5–14 days) and mature milk (between 15 days and 12 months postpartum). Ten studies were of longitudinal design [29,34,35,38,39,40,41,42,43,45] and three were cross-sectional [36,37,44]. In one cross-sectional study, the majority of participants provided milk samples/were measured at one time point, at either 1, 2, or 3 months postpartum, but approximately 15% of dyads were investigated at two time points (reported by the authors on request) [44].

### 3.3. Determination of 24 h Milk Intake

The majority of studies (12/13) used the method of test-weighing the infant (or, in one study, the mother [29]) pre- and post-feed [24] to estimate 24 h MI, of which two studies were corrected for insensible water loss [29,45]. One study indirectly estimated 24 h MI using the dose-to-the-mother deuterium-oxide (^2^H_2_O) turnover method [34]. Within these seven cohorts, during stable lactation [22] (measured around 3 months in most of the selected studies), the mean 24 h MI ranged from 727 ± 164 to 891 (groups’ combined mean) and sample size ranged from 11 to 71.

### 3.4. Methodology of Human Milk Sampling and Storage

The time and technique of HM sample collection differed between the studies. The following sampling procedures were reported: pre-/post-feed sampling [29,35,36,37,38,40,41,42,43], pre-feed sampling only [39], post-feed sampling only [34], mid-stream sampling [44], and alternate breast expression [45].

Collection of the samples throughout the day also varied, with 7/13 studies reporting predominantly morning collection [35,38,40,41,42,43,44]. Studies also reported sampling over a 24 h period [29,45], morning and evening sampling [39], and sampling at least two hours after the breastfeed or expression [36,37], while one study did not specify the time [34].

HM was hand-expressed into sterile tubes either aseptically [36,37] or non-aseptically [35,38,39,40,41,42,43], or collected using a combination of hand and/or breast pump expression [29,34,44]. One study did not specify if breast expression was performed with the aid of a pump and/or by hand [45].

Upon collection, samples were either initially stored in a participant’s household freezer for a maximum of 24 h [29,39] or frozen in a research room freezer at −20 °C [35,38,40,41,42,43], and transported on ice to the laboratory, where samples were stored at either −20 °C [29,35,38,40,41,42,43,44] or −80 °C [39] until analysis (reported by the corresponding authors on request). Samples were also stored at 4 ℃ in the participant’s household fridge for 6–24 h and later transported on ice to the laboratory, where they were stored at −80 °C [36,37], or samples were collected at the research facility and kept frozen at −70 °C [34], until further analysis. One study did not clearly specify the storage/transit conditions [45].

### 3.5. Methodology of Human Milk Analysis

HM total protein, casein, and whey protein were measured using the Bradford protein assay [29,35,38,42] and the Kjeldahl and Lowry method [44,45]. Protein concentration was also calculated from nitrogen measured by the DUMAS method [34].

Lactose was measured with enzymatic spectrophotometry (modified Kuhn and Lowenstein method) [29,35,37,38,41], ^1^H-nuclear magnetic resonance (NMR) [34], and the modification of the lactase assay method described by Dahlqvist [45] (the latter lactose results were used only for the energy intake calculation). Glucose was quantified by enzymatic assay [37]. Total carbohydrates were measured using the mid-infrared spectroscopy and UV spectrophotometry [35,38,41].

Fat was measured by the van de Kamer method [44], using the modified colorimetric spectrophotometric method of Stern and Shapiro [29] and the modified Folch extraction procedure [45] (the latter lipid results were used only for the energy intake calculation). Triglycerides were measured by NMR [34] and milk fat globule membrane (MFGM) lipids by mass spectrometry [39].

The enzyme-linked immunosorbent assay (ELISA) was used to quantify adiponectin [35,38,43,44], ghrelin and IGF-1 [44], insulin [37], lactoferrin [35,38,40], sIgA [35,38,40], skim milk leptin [43,44], and whole milk leptin [35,38,43].

Lysozyme was measured using a modified Selsted and Martinez method [35,38,40].

Nineteen individual HMOs were identified and quantified using high-performance liquid chromatography (HPLC) [36]. Total HMO concentration was calculated by subtracting the concentration of lactose from the total carbohydrate concentration [41].

### 3.6. Methodology of Measuring Infant Body Composition and Growth Parameters

Infant anthropometric measurements included length, weight, body mass index (BMI), and head circumference, and were converted to BMI-for-age (BMIAZ), length-for-age (LAZ), weight-for-age (WAZ), and weight-for-length (WLZ) z-scores [36,37,44], or to sex- and age-adjusted standard deviation scores (SDSs) [34].

Infant total body water using the isotope-dilution method [45], fat-free mass (FFM) with bioelectrical impedance spectroscopy (BIS) [36,37], or both, FFM with BIS and percent fat mass (%FM) with ultrasound skinfolds (US: US2SF, triceps, subscapular; US4SF, biceps, triceps, subscapular, and suprailiac) [40,41,42,43] were estimated. Whole BC parameters, including lean body mass, FFM, FM and/or percentage FM (%FM), the height-normalised FM index (FMI) and FFM index (FFMI), and FM to FFM ratio (FM/FFM), were further calculated.

Three studies investigated infant regional BC, including skinfold thickness (triceps, subscapular, flank, and quadriceps) [34], visceral and subcutaneous-abdominal fat areas [38], and mid-arm and mid-thigh fat and lean areas [35].

### 3.7. Statistical Analysis

For the investigation of relationships between intakes of HM component and infant BC outcomes, statistical approaches included linear regression models [29,34,36,37,40,41,42,43,45], linear mixed models [34,35,38,40,41,42,43], and Pearson’s correlation analyses [39,45]. One study used Pearson’s correlation to analyse normally distributed variables and Spearman’s correlation for variables not normally distributed (reported by the authors on request) [44].

Studies were adjusted for several potential confounders, including infant age [34,35,38,40,41,42,43], birth weight [34,37,45], sex [34,45], gestational age [34], feeding mode [34], size at the beginning of each time interval [45], previous gain [45], mean parental height [45], maternal percent ideal body weight [45], season [45], hours in day care [45], and both maternal secretor status and interaction between secretor status and predictor [36].

Seven studies, which included six studies conducted on the same cohort, accounted for multiple comparisons [34,35,38,40,41,42,43].

### 3.8. Risk of Bias

**Figure 2:**
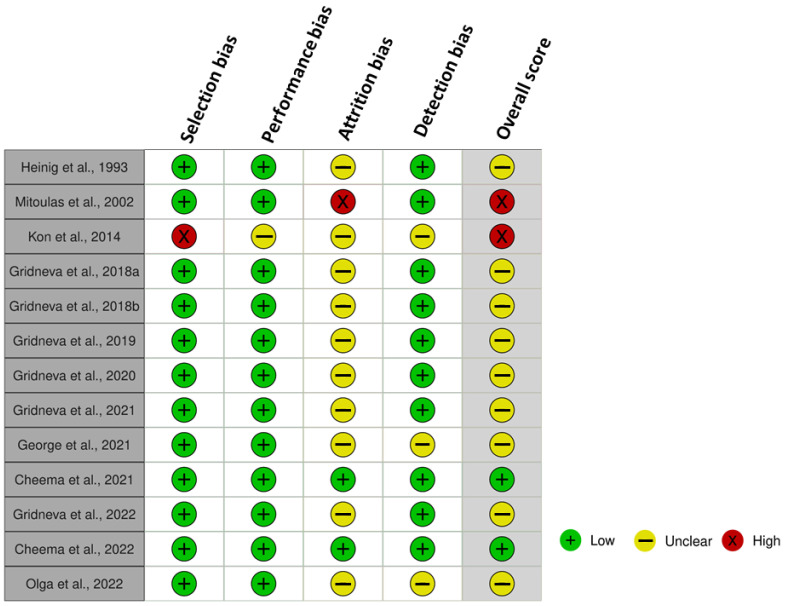
Risk of bias in studies [29,34,35,36,37,38,39,40,41,42,43,44,45] assessing the relationship between intakes of human milk components and infant body composition and anthropometry using the National Institute for Clinical Excellence methodological checklist.

The studies included in this review were evaluated for risk of bias and overall quality using the NICE methodological checklist, with the majority rated as medium quality (70%, 9/13) (Figure 2). High risk of bias was detected in 15% (2/13) of the studies. The main issues identified were attrition bias and lack of adjustment for potential confounders (detection bias).

### 3.9. Twenty-Four-Hour Intakes of Macronutrients and Infant Body Composition

**Figure 3:**
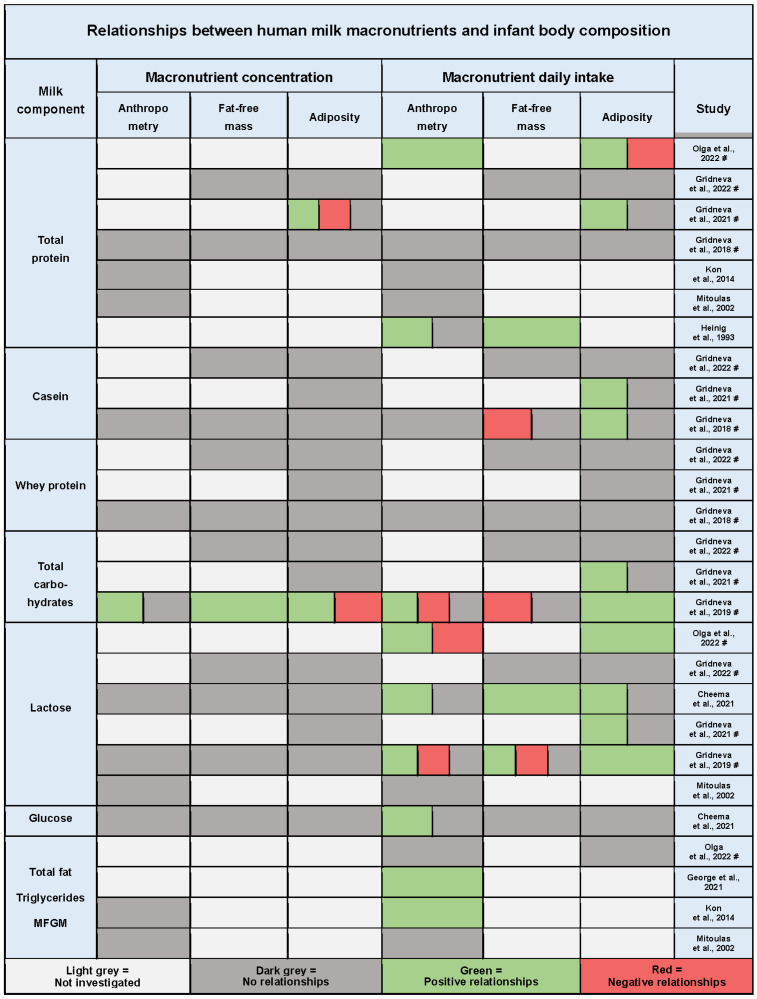
Summary of the results of quantitative synthesis for studies investigating relationships between human milk macronutrient intake and infant anthropometry, fat-free mass, and adiposity. Each cell represents one or multiple significant or non-significant results reported by the study. Significance level was determined by the study and, where multiple comparisons adjustment was performed (indicated by #), only results significant after the adjustment are presented; if no adjustment was performed, the results for *p* < 0.05 are presented. MFGM, milk fat globule membrane lipid species [29,34,35,37,38,39,41,42,44,45].

Of 13 eligible studies, 10 assessed relationships of infant BC and growth outcomes with HM macronutrients and analysed 173 HM components, including 166 MFGM lipids species (Figure 3, Table A1). The short summary of macronutrient intakes grouped by time postpartum are presented in Table A2. The 24 h intakes of total protein and lactose were comparable during stable lactation (1–6 months) between studies; however, small sample sizes and heterogeneity in data reporting made any formal comparison not prudent.

Significant associations with infant anthropometrics and BC were found for intakes rather than concentrations of most HM macronutrients. Only two studies reported concentration associations that, to some degree, matched the intake associations for total protein [38], yet were opposite for total carbohydrates [41].

There was greater agreement between the studies that investigated intakes of macronutrients, with 15 relationship matches. The direction of multiple reported relationships of macronutrients with early infant BC outcomes was predominantly positive for infant adiposity (1/10 studies reporting positive relationships also reported negative) and anthropometrics (3/9 studies reporting positive relationships also reported negative), while no consistent pattern was observed for macronutrient intakes and infant FFM (Figure 3).

#### 3.9.1. Protein

Total protein was the most frequently studied HM macronutrient (7/10 studies, Figure 3, Table A1 and Table A2). Four out of seven studies (57%) found no significant relationships between total protein intake and infant anthropometry or BC [29,35,42,44]. Three studies found positive relationships with infant anthropometry [34,45], FFM [45], and adiposity [34,38]; however, one of these studies also reported a change in the direction of the relationship with infant adiposity (skinfold gain) from positive to negative from 3 months of age onwards [34].

Three studies reported on the effect of HM casein intake and concentration on infant anthropometry and BC [35,38,42]. Casein intake was positively associated with infant whole body adiposity (FM, %FM, and FMI) [42] as well as subcutaneous-abdominal adiposity [38] and negatively with FFM [42], but not with mid-arm and mid-thigh lean and fat areas [35]. The same three studies also analysed the effect of whey protein on infant BC outcomes and found no relationships after accounting for multiple comparisons.

#### 3.9.2. Carbohydrates

Six out of ten studies analysed relationships between HM lactose intake and BC outcomes [29,34,35,37,38,41] (Figure 3, Table A1). Most studies (67%, 4/6) found predominantly positive relationships between lactose intake and infant anthropometry [34,37,41], FFM [37,41], and adiposity parameters [34,37,38,41]. However, two studies reported a change in the direction of the relationship, similar to protein, from positive to negative. Negative relationships were found with infant BMI and FFMI between 5 and 12, at 12 months of age [41], and with weight gain after 3 months of age onwards [34]. No relationships with the concentration of lactose were shown.

In addition to lactose, three studies from the same cohort analysed associations between 24 h intake of HM total carbohydrates and infant anthropometry and BC with contradicting results. One study of the two that investigated regional BC did not find any associations with infant lean and fat limb areas [35], while a study of infant subcutaneous-abdominal adiposity reported positive associations with total carbohydrate intake [38]. The third study investigated whole BC and reported opposite associations for intakes and concentrations of total carbohydrates [41]. Infant FM associations were positive with total carbohydrate intake and negative with concentration, yet for FFM, the associations were negative with intake and positive with concentration.

Only one study investigated relationships between HM glucose intake and infant BC outcomes, reporting that a higher glucose intake was associated with an increased head circumference [37].

#### 3.9.3. Fat

Four studies analysed 24 h HM fat intake relationships with infant anthropometry and BC (Figure 3, Table A1). Two longitudinal studies that measured total fat intake did not report any relationships with either anthropometry [29,34] or skinfold gains [34]. The third longitudinal study that assessed intakes of individual MFGM lipid species found a large number of time-dependent positive correlations for 99 of the 166 measured species with infant weight, head circumference, and WLZ, with head circumference relationships being the strongest [39]. Cross-sectionally, at 3 months postpartum, a higher 24 h total fat intake was found among high weight gain infants compared with low weight gain infants [44].

Studies assessing fat concentration in addition to intake did not establish any relationships with BC or growth [29,44]. It is of note that only one study calculated fat intake by sampling HM pre-/post-feed over 24 h [29], with the other three studies estimating fat intake based on two (morning and evening) pre-feed samples [39] or a single mid-stream [44] or post-feed sample [34]. As fat concentration progressively increases during a breastfeed [50], the results are unlikely to be representative of true intake, as demonstrated by George et al. [51].

### 3.10. Twenty-Four-Hour Intakes of Bioactive Molecules and Infant Body Composition

**Figure 4:**
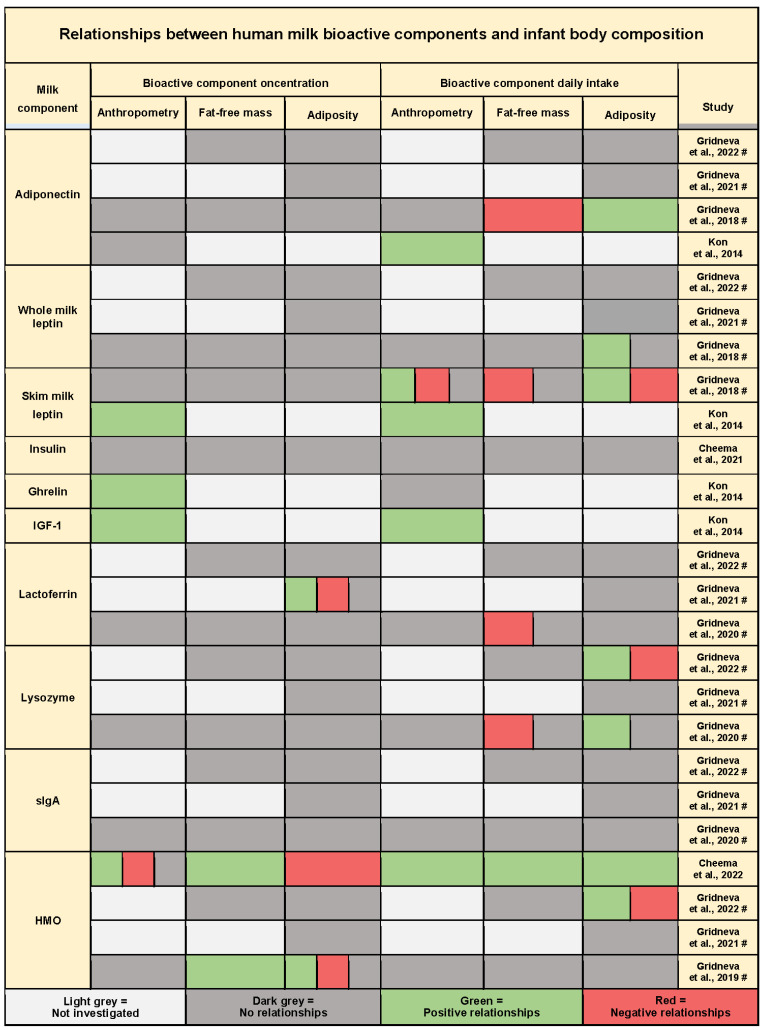
Summary of the results of quantitative synthesis for studies investigating relationships between intake of human milk bioactive components and infant anthropometry, fat-free mass, and adiposity. Each cell represents one or multiple significant or non-significant results reported by the study. Significance level was determined by the study and, where multiple comparisons adjustment was performed (indicated by #), only results significant after the adjustment are presented; if no adjustment was performed, the results for *p* < 0.05 are presented [35,36,37,38,40,41,43,44].

Eight studies assessed the relationships of infant BC with HM bioactive molecules and analysed 28 components, including metabolic hormones, immunomodulatory proteins, and total and 19 individual HMOs (Figure 4, Table A3). The short summary of bioactive molecule intakes grouped by time postpartum is presented in Table A4. Small sample sizes and heterogeneity in analytical methods and data reporting made any formal comparison not prudent.

Time-dependent relationships with infant anthropometrics and BC were found for intakes and not for concentrations of several HM bioactive molecules, such as adiponectin, whole milk leptin, and lysozyme. Only four studies that investigated intakes of bioactive molecules reported concentration relationships, three of which did not match the intake results.

The results for intakes of bioactive molecules were less uniform than those of macronutrients, with a smaller number of studies focusing on the same HM component. The directions of the three relationships matched between the studies for intakes of skim milk leptin [44,49], lysozyme [35,40], and HMOs [35,36]. One match between the studies for HMO concentration was also shown [36,41]. The direction of multiple reported relationships of bioactive molecules with infant BC outcomes was predominantly positive for infant adiposity (3/7 studies reporting positive relationships also reported negative) and anthropometrics (1/5 studies reporting positive relationships also reported negative), while the reported relationships with infant FFM were predominantly negative (4/5) (Figure 4).

#### 3.10.1. Metabolic Hormones

Positive relationships between intakes of metabolic hormones and infant BC and anthropometry were found in several studies (Figure 4, Table A3).

Four studies investigated 24 h intake of HM adiponectin [35,38,43,44]. Cross-sectionally, at 3 months postpartum, skim milk adiponectin intake was higher in infants with high weight gain compared with the low weight gain group [44]. In a longitudinal cohort, whole milk adiponectin intake was significantly associated with infant whole BC, positively with adiposity, and negatively with FFM [43]. After adjusting for multiple comparisons, no significant associations with infant regional BC (visceral and subcutaneous-abdominal adiposity [38], as well as mid-arm and mid-thigh lean and fat areas [35]) were found.

The four studies that investigated adiponectin also measured HM leptin [35,38,43,44]. Two of the studies, which measured leptin in whole milk, found daily intakes were positively associated with infant whole body adiposity (higher leptin intakes at 12 months were associated with increased changes in infant FM and %FM between 2 and 12 months) [43]. No association was shown with visceral and subcutaneous-abdominal adiposity [38]. One study measured leptin in both whole and skim milk, with multiple skim milk leptin associations contrasting the whole milk leptin results [43]. In the cross-sectional study, higher skim milk leptin intakes were found in infants with high weight gain compared with the normal weight gain group at 2 months, while skim milk leptin concentrations were significantly higher in milk consumed by the infants with normal weight gain compared with the low weight gain group at 3 months of lactation [44].

The cross-sectional study was also the only one to assess HM IGF-1 and found, at 3 months postpartum, milk with a higher IGF-1 concentration was consumed by infants with high weight gain compared with the low weight gain group, and higher intakes IGF-1 were found in the groups of infants with high and normal weight gain compared with the low weight gain group [44].

The same study [44] also reported that, at 1 month postpartum, HM ghrelin concentrations were significantly higher in milk consumed by infants with high weight gain compared with those with normal weight gain, and by infants with normal weight gain compared with those with low weight gain. However, there was no difference between groups by infant intake of ghrelin at any lactation time points.

HM insulin intake was also assessed by one study, reporting no relationships with infant BC or anthropometry [37].

#### 3.10.2. Immunomodulatory Proteins

Three studies from the same cohort analysed relationships between intakes of HM lactoferrin, lysozyme, and secretory immunoglobulin A (sIgA) and infant BC development during the first 12 months of lactation [35,38,40] (Figure 4, Table A3).

Higher HM lysozyme intake during the first 12 months of lactation was associated with increased whole body adiposity (FM, FMI), while higher lysozyme intake at 12 months of age was associated with a decrease in infant FFMI between 5 and 12 months [40]. The association of lysozyme intake with infant mid-arm fat areas was time-dependent, positive at 2, 5, and 9 months of age and negative at 12 months [35]. After adjusting for multiple comparisons, no association was reported between lysozyme intake and visceral and subcutaneous-abdominal fat areas [38] or between lysozyme concentration and whole BC [40].

Increased HM lactoferrin intake during the first 12 months was associated with a decrease in infant FFMI [40], with no significant relationships with infant regional adiposity [35,38] or regional lean mass [35]. Lactoferrin concentration was positively associated with infant visceral depth at 5 and 9 months and negatively at 2 and 12 months [38].

After adjusting for multiple comparisons, no significant associations with either intake or concertation of HM sIgA were reported [35,38,40].

#### 3.10.3. Human Milk Oligosaccharides

Four studies investigated either combined total (calculated as a subtraction of lactose concentration from total carbohydrate concentration) [35,38,41] or multiple individual HMOs [36] and found relationships with infant BC (Figure 4, Table A3).

Three studies from the same longitudinal cohort analysed relationships between intakes of total HMOs and infant whole [41] and regional [35,38] BC. After adjusting for multiple comparisons, no associations between intake of total HMOs and infant FFM or FM parameters were found, yet a higher total HMO concentration was associated with greater infant FFM and FFMI, and decreased FMI, %FM, and FM/FFM ratio at 5, 9, and 12 months (increased at 2 months) [41]. Total HMO intake did not relate to infant visceral and subcutaneous-abdominal adiposity [38], but was time-dependently associated with infant mid-arm fat area [35], negatively at 2 and positively at 5, 9, and 12 months of age.

In a recent larger cross-sectional study, positive associations were shown between intakes of six individual HMOs (2′-fucosyllactose (2′FL), 3-fucosyllactose (3FL), difucosyllactose (DFLac), difucosyllacto-N-hexaose (DFLNH), difucosyllacto-N-tetrose (DFLNT), and sialyl-lacto-N-tetraose (LSTb)) and multiple infant BC (FFM, FM) and anthropometry measures [36]. However, while the direction of intake relationships was positive, the concentration relationships did not match the intakes for all but one HMO (DFLNH), being either absent (2′FL and DFLac) or negative (ucosyllacto-N-hexaose (FLNH), lacto-N-neotetraose (LNnT), and lacto-N-fucopentaose III (LFNP III)). Additionally, relationships with infant BC were dependent on maternal secretor status [36].

## 4. Discussion

**Figure 5:**
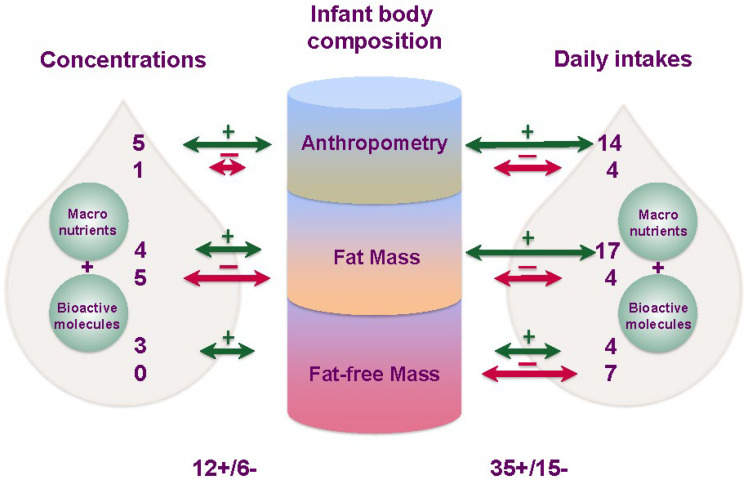
Summary of multiple significant relationships between infant anthropometry and body composition and concentrations and daily intakes of human milk (HM) components. Relationships are indicated by arrows (green—positive; red—negative), while numbers represent the quantity of studies that have reported at least one positive or negative relationship.

This is the first systematic review that has summarised the findings on relationships between the 24 h intake of HM macronutrients and bioactive components and infant BC and growth outcomes. The data evaluated showed significant time-dependent relationships between 24 h intake of HM components and infant BC. In particular, studies demonstrated predominantly positive relationships with infant adiposity and negative relationships with FFM for intakes, but not for concentrations of HM components (Figure 5). These data further emphasise the importance of measuring the intake of HM components in addition to concentration, which may be misleading when investigating the relationships of HM components with infant outcomes. The findings of this review also suggest that the body of literature related to HM and infant BC be viewed through the lens of the measures of milk employed for the study.

Despite several decades of research on HM composition and its effect on infant growth, and a recent change of research focus from anthropometry to infant BC, few studies comprehensively investigated the effects of intake of HM components on infant growth outcomes. There is, however, some recognition in the research fields of infant formula and preterm infants, where MI can be more readily measured. This contrasts with the difficulties encountered with measuring breastfed infant 24 h MI in the cohort setting, particularly at later months of lactation. Completing a 24 h MI measurement, especially with pre- and post-feed sample collection, is somewhat labour-intensive for mothers. Nevertheless, it provides crucial information on any HM component that cannot be obtained from a single milk sample, which only allows for concentration analyses.

This systematic review demonstrated distinct patterns of disparity between reported relationships of intakes and concentrations of HM components. Most studies reported either no relationships with concentrations where intakes were significantly related, or occasional opposing relationships for both HM macronutrients and bioactive molecules (Figure 3 and Figure 4). Only five significant relationship matches between concentrations and intakes of the same HM component were found. While there were 18 significant relationship matches between studies of intakes, there was only 1 match for concentration relationships, indicating the true potential value of the intake approach. The components in the greatest agreement were total protein, lactose, and HMOs.

The evidence of relationships of intake of HM components with infant BC remains limited owing to a lack of studies and heterogeneity in study design, milk analytics, BC methodology, and statistical approaches. Measurement of infant MI is possible via two validated methods, test-weighing and deuterium dilution studies. These two methods are non-invasive and thus do not affect milk production or disturb feeding patterns, are highly correlated with validation standards [52], and tend to be closer to each other when deuterium is administered to mothers (as opposed to infants) and when test-weighing values are corrected for insensible water loss [28]. However, both methods heavily rely on participant compliance and with deuterium dilution cost (especially when given to mother) and the availability of isotope may be a limitation for use in many research settings, while with test-weighing, high accuracy electronic scales are needed [52]. The majority of the reviewed studies employed test-weighing, with one study using the dose-to-the-mother deuterium-oxide (^2^H_2_O) turnover method [34], which does not account for supplementary feeds if given [53]. As 24 h MI did not differ substantially between the selected studies and was similar to reference studies in healthy cohorts [22,54], the studies were unlikely to include subjects with low milk production. Another strength is that the majority of the studies were longitudinal, with HM sampling and infant growth parameters measured at multiple time points.

HM sampling techniques and compositional analyses, however, showed heterogeneity between studies of the same HM component, particularly HM fat. Fat is the most variable HM component within and between breastfeeds and throughout the 24 h and lactation [55], as its concentration relates to the degree of breast fullness [22,56] and maternal diet [57,58]. This makes it difficult to confidently measure fat intake, particularly if only one sample is collected. Mitoulas et al. [29] used the optimal approach, sampling before and after each breastfeed over 24 h, and used a colorimetric spectrophotometric method. George et al. [39] utilised mass spectrometry, but collected morning and evening pre-feed samples and reported on MFGM lipid species, which account for up to 2% of total HM fat. Kon et al. [44] collected single mid-stream milk sample and used van de Kamer method designed for estimation of fecal fatty acids [59] without mentioning modification for HM. Olga et al. measured triglycerides by NMR [34], but sampled after a breastfeed and, as post-feed samples usually have higher fat concentrations [50], the intakes calculated from these concentrations may not be representative of actual infant fat intakes. George et al. reported either a mean fat intake underestimate greater than 8 g/day (close to a third of the true total daily intake) or a mean fat intake overestimate of 18 g/day (which is more than half of the true daily intake) depending on the sampling protocol [51]. It is not surprising that no consensus on the fat intake effect was reached.

Another example of method heterogeneity is HM leptin. The majority of HM leptin studies are conducted on skim HM. As the concentration of whole milk leptin is significantly higher than that of skim milk leptin, with no apparent direct relationships between them, or with either the volume of milk removed or fat concentration [60], the results for skim milk leptin should be interpreted with caution, as they do not represent the true amount of leptin ingested by infant. Both studies that analysed skim milk leptin intake [43,44] found relationships with infant anthropometry and BC, yet studies of whole milk leptin intake [35,38,43] did not support these findings. HM leptin is widely studied and its concentration relates positively to maternal adiposity [61,62]. Despite no firm evidence, it is speculated that infants of mothers with obesity (and higher HM leptin concentrations) are likely to consume more leptin, which may negatively impact their growth [63]. Measurement of MI and intake of leptin need to be carried out; however, this will likely be fraught with additional lactation issues experienced by women with obesity, such as low milk supply, leading to supplementation [64,65].

Additionally, differences in methods of measuring infant BC could influence the results. Infant anthropometric measurements such as length, weight, and BMI are usually collected, and three studies were restricted to anthropometry only [36,37,44]. Recently, it became apparent that not only quantity, but also the quality of infant growth is important, supporting the need for BC measurements. BC is a good indicator of infant growth and development and of nutritional adequacy, and is a more reliable predictor of some health outcomes than anthropometric measurements. None of the studies used reference BC methods, such as whole-body air-displacement plethysmography (ADP) or dual-energy X-ray absorptiometry (DEXA) [66], or focused on breastfed infants’ skeletal development (bone density), which is known to be affected by nutrition type [67,68]. This is likely because of both cost and issues with the use of reference methods in paediatric participants, such as exposure to low levels of radiation, requirement of the infant to be restrained, or involving repeated blood sampling. It is noted that low-cost, simple-to-use BC methods usually exhibit the lowest accuracy and precision; however, they are more readily applied in a small to medium cohort setting.

Out of 10 studies that evaluated infant BC, only 1 study used the isotope-dilution method to measure infant total body water [45]. This method is considered accurate; however, it is also subject to error if used alone as a two-compartment method owing to variations in the infant FFM composition, especially in the case of over- or under-hydration. In six studies, infant whole BC was measured with less accurate but transportable and easy to use BIS [36,37,40,41,42,43], applying validated age-appropriate BIS BC prediction equations [69,70]. Four of these studies [40,41,42,43] additionally used BC equations that utilised ultrasound skinfolds measurements [70], showing some similarity in the results between the two methods. Three studies investigated infant regional BC and measured skinfold thickness with calipers [34], mid-arm and mid-thigh fat and lean areas with ultrasound [35], and visceral and subcutaneous-abdominal fat areas with ultrasound [38]. Despite variability in the methods, some degree of consistency in the results was observed within and between studies and cohorts, particularly for macronutrient intakes (Figure 3).

Finally, statistical approaches differed between studies. Either linear regression models or linear mixed models were used by the majority of studies (12/13), with more than half (7/13) adjusting for infant age. However, accounting for multiple comparisons, which is currently frequently requested by reviewers, makes the comparison and meta-analysis of these recent and historical data challenging.

With the low number of studies available and a wide focus on multiple HM components, it is difficult to summarise the findings for particular components, especially where the results could be impacted by the methodology, such as with leptin and fat intake. However, there are some similarities of note between the findings of several studies in HM components, particularly macronutrients.

Total protein was the most frequently studied, with 7/10 macronutrient studies measuring protein intake. Three studies from three cohorts reported predominantly positive relationships with infant anthropometry [34,45], FFM [45], and adiposity [34,38] (Figure 3). This is not surprising, as protein plays an important role in the programming of infant growth and adiposity. Indeed, multiple clinical trials and observational studies have shown that lowering the protein content in infant formula may reduce the risk of developing obesity later in life [17]. Lower protein intake from HM (compared with cow’s-milk-based formula) may be one of the protective factors of breastfeeding that lead to a reduction in obesity risk, the effect known as early protein hypothesis, while higher protein intake results in faster weight gain in infancy, which is in turn associated with increased adipogenesis and later obesity risk [17]. While three studies found positive relationships, two studies [34,42] also indicated there may be a change in the direction of the total protein intake relationship with infant BC, from positive to negative, as lactation progresses (though rendered insignificant after multiple comparisons adjustment in one [42]). Further, a third study did not differentiate between HM protein intake and intake of protein from supplementary foods after 3 months of age [45]. This suggests that HM total protein, which is a mixture of over 400 proteins [71], many of which have immunological and metabolic functions, may create a favourable environment for growth and time-specific programming of infant BC development. This is further supported by casein intake relationships, which are associated positively with infant adiposity, but negatively with FFM, with whey protein also displaying similar relationships prior to adjustment for multiple comparisons [42].

HM lactose intake was measured in four cohorts (6/10 studies) and was predominantly positively related to infant anthropometry and adiposity (Figure 3). Lactose is the least variable HM component [72], contributing to approximately 44% of HM energy content [73]. Thus, the reported strong positive relationships with infant BC are somewhat expected. There was no strong agreement on relationships with infant FFM, though the results indicate a time-dependent effect. The cross-sectional cohort reported positive lactose intake relationships with infant FFM and FFMI at 3 months of age [37]. The longitudinal cohort established positive relationships with FFMI between 2 and 9 months and negative at 12 months of age [41]. Similar to total protein intake, lactose intake also displayed time-dependent relationships with infant anthropometry and BC, with a change to the negative direction of the relationship in two cohorts, after 3 [34] to 5 months of age [41].

Unlike HM macronutrients, HM bioactive component intakes are not well investigated, with only leptin (discussed above), adiponectin, and HMOs being reported by two cohorts and the rest of the bioactive component intakes being measured in one cohort only (Figure 4). HMOs are the third-most abundant class of bioactive molecules in HM [74], and HMO intakes and infant BC were investigated in two cohorts, with contradicting results. The longitudinal cohort found predominantly negative relationships between total HMO intake and whole BC that were rendered insignificant after multiple comparisons adjustment [41] and reported no associations with infant abdominal adiposity [38]. However, total HMO intake was time-dependently associated with infant mid-arm fat area, negatively at 2 months of age and positively at 5, 9, and 12 months [35]. Yet, in the cross-sectional cohort, strong positive associations between intakes of six individual HMOs and infant anthropometry, FFM, and adiposity were found at 3 months of age [36]. This may be owing to the methodology and the increased number of dyads included in the cross-sectional study compared with the longitudinal study. Further, there are plausible mechanisms by which HMOs may impact infant growth, such as enhancing the growth of beneficial bacteria in the gut and altering the structure or function of the gut microbiome during the periods critical for programming infant BC [75,76].

Relationships of HM adiponectin intake and infant anthropometry also differed between the longitudinal [43] and cross-sectional [44] cohorts. Infant BC was not analysed in the latter study, yet the longitudinal study reported positive relationships with infant adiposity and negative with FFM. Whole milk leptin intake was also positively related to infant adiposity [43] and intake of IGF-1 to infant weight gain, but both HM insulin intake and intake of leptin antagonist, ghrelin, were not implicated in infant BC [37,44]. This could be owing to sampling mid-stream and measuring in skim milk, as ghrelin concentration is known to be reduced in post-feed samples [77] and in skim milk [78].

Only one cohort investigated the relationships of 24 h intakes of immunomodulatory proteins such as lactoferrin, lysozyme, and sIgA with infant whole and regional BC [40], reporting either positive or time-dependent relationships with infant adiposity for lysozyme and negative with FFM for both lysozyme and lactoferrin. As a substantial part of HM whey and total protein fractions, these HM immune factors may exert time-specific effects on infant BC and growth, and their importance is reflected by the fact that their concentrations increased from the 2nd to 12th month of lactation, providing stable intakes of these components by the infant throughout the first year of life [40].

In summary, this first systematic review of its kind has established that, despite decades of research in HM composition, there is a scarcity of studies investigating the impact of the actual intake of HM components on infant growth and BC. While HM components’ concentrations are essential to determine the mother–milk communication within the co-adapting breastfeeding dyad, evaluating concentrations alone without measuring infant MI may provide misleading results. Importantly, interventions designed to improve infant growth and health should consider HM intake.

These limited data indicate predominantly positive relationships between intakes of HM macronutrients and infant growth and BC parameters, while relationships of intakes of bioactive molecules are less uniform. Further, relationships between HM macronutrients and bioactive components intakes and infant adiposity are predominantly positive, while those between intakes of bioactive components and infant FFM are predominately negative. Furthermore, intake relationships are often time-dependent, with changes in the direction of the relationship observed for some HM components as lactation progresses. Positive relationships in early lactation months and negative in later lactation were reported by two studies for lactose and total protein intake [34,41]. Five studies showed a similar change in the direction of relationships prior to multiple comparisons adjustment for intakes of lactose, total protein, casein, adiponectin, whole and skim milk leptin, lysozyme, and sIgA [34,40,41,42,43]. This observed change in relationships later in lactation could be due to smaller amounts of solid foods consumed when MI is larger; however, only one study measured the infant diet from the beginning of weaning [45] without further report.

This review calls for more larger well-designed studies that can take a holistic approach, treating HM as a complex and diverse biological matrix, thus including multiple HM components and their intakes to determine how HM meets the nutritional and developmental requirements of the infant. It is encouraging that some recent studies [34,46,47,48] have focused on HM component intakes, with a promise of more high-quality research from these research groups in the future, which will be welcomed by academics and health professionals working to improve maternal and child health.

## 5. Conclusions

Accumulating evidence indicates that intakes of HM macronutrients and bioactive components are implicated in the development of infant BC and growth. However, with the limited number of studies as well as heterogeneity in study designs, it is difficult to draw firm conclusions regarding the direction of the associations of some HM components with infant BC and growth parameters. The findings of this review suggest that measuring the concentration of HM components without quantifying their intake by the infant may be excluding an important mechanism by which HM confers infant health benefits. Future studies should measure the 24 intake of HM components to further elucidate their effects on infant growth and BC development.

## Data Availability

Data sharing not applicable.

## Supplementary Materials

The following supporting information can be downloaded at https://www.mdpi.com/article/10.3390/nu15102370/s1. Tables S1–S4: Search strategy for infant intake of human milk components and infant body composition and growth in MEDLINE, EMBASE, and CINAHL PLUS.

## Appendix A

**Table A1:** Summary of studies investigating the relationships between intakes of human milk macronutrients and infant anthropometrics and body composition.

Author, Year, Country	Study Type, Time Postpartum, Sample Size	Sample Type, Collection, Analyses	24 h Milk Intake and Body Composition Methods	Component Concentration	Component 24 h Intake	Relationship with Anthropometry or Body Composition	ß (SE) or *r*	*p*-Value
Olga et al., 2022 United Kingdom [34]	LS, Birth–12 m, n = 94 (complete measurements *n* = 47) EBF to 6 w + (*n* = 70) EBF to 3 m + (*n* = 60) EBF to 6 m + (*n* = 22)	SHM/WHM, post-feed sampling DUMAS method (total nitrogen for protein calculation), ^1^H-NMR (carbohydrates and triglycerides)	4–6 w: 780 ± 160 mL/24 h (450–1260) (*n* = 70) EBF at 3 m, 4–6 w: 800 ± 160 mL/24 h (*n* = 60) 24 h MI: dose-to-the-mother deuterium-oxide (^2^H_2_O) turnover BC: anthropometry and skinfold thickness (triceps, subscapular, flank, quadriceps)	(*n* = 59) Carbohydrates, kcal/100 mL 6 w: 25.1 ± 1.5	(*n* = 47) Carbohydrates, g/24 h 6 w: 50.8 ± 11.7	No concentrations and BC analysis	NR	NR
						Carbohydrate intake at 6 w		
						Weight gain: ↑0–6 w;	0.04 (0.01) ^a^	<0.001 ^b^
						↓3–12 m	–0.03 (0.01)	0.025 ^b^
						BMI gain: ↑0–6 w	0.05 (0.01)	0.002 ^b^
						Skinfold gain: ↑0–6 w;	0.06 (0.01)	<0.001 ^b^
						↓3–12 m	–0.04 (0.01)	0.009
						Carbohydrate intake and SDS 0–3 m		
						↑Weight SDS	0.02 (0.01)	0.002 ^b^
						↑BMI SDS	0.02 (0.01)	0.008 ^b^
						Carbohydrate intake and SDS 3–12 m		
						↓Weight SDS	–0.02 (0.01)	0.030
						↓Length SDS	–0.02 (0.01)	0.040
				Protein, kcal/100 mL 6 w: 4.6 ± 0.9	Protein, g/24 h 6 w: 9.1 ± 2.1	Protein intake at 6 w		
						Weight gain: ↑0–6 w	0.22 (0.04)	<0.001 ^b^
						BMI gain: ↑0–6 w	0.22 (0.08)	0.010
						Skinfold gain: ↑0–6 w;	0.28 (0.07)	<0.001 ^b^
						↓3–12 m	–0.19 (0.07)	0.006 ^b^
						Protein intake and SDS 0–3 m		
						↑Weight SDS	0.05 (0.02)	0.010
						↑BMI SDS	0.06 (0.03)	0.040
						Protein intake and SDS 3–12 m		NS
				Fat, kcal/100 mL 6 w: 35.3 ± 19.0	Fat, g/24 h 6 w: 29.6 ± 17.4	Fat intake at 6 w		
						Length gain: ↓0–6 w;	–0.02 (0.01)	0.020
						↑6 w–3 m;	0.01 (0.01)	0.010
						BMI gain: ↑0–6 w	0.03 (0.01)	0.020
						Fat intake and SDS 0–3 m		NS
						Fat intake and SDS 3–12 m		NS
Gridneva et al., 2022 Australia [35]	LS, 2–12 m, *n* = 20, 2 m (*n* = 14) 5 m (*n* = 20) 9 m (*n* = 18) 12 m (*n* = 18) EBF to 5 m	SHM, pre-/post-feed, morning sampling ^d^ Bradford assay (casein, total, and whey protein), enzymatic spectrophotometry (Kuhn and Lowenstein, lactose), deproteinisation with trichloroacetic acid, dehydration by sulfuric acid (TCH)	5 m: 819 ± 205 (498–1185) (*n* = 17) 9 m: 478 ± 154 (300–775) (*n* = 8) 12 m: 451 ± 216 (255–795) (*n* = 8) 24 h MI: pre-/post-feed test weighing infant Regional BC: US scans of anterior upper arm and thigh	Total protein, whey protein and casein in Gridneva et al., 2018 (a) [42] below TCH and lactose in Gridneva et al., 2019 [41] below	Total protein, whey protein and casein in Gridneva et al., 2018 (a) [42] below TCH and lactose in Gridneva et al., 2019 [41] below	All macronutrient intakes (overall)	NR	NS
						Total protein concentration		
						Mid-thigh lean area: ↓2, 5 m, ↑9 m, ↓12 m	NA ^e^	0.030 ^c^
						Whey protein concentration		
						Mid-thigh lean area: ↓2, 5 m, ↑9 m, ↓12 m	NA ^e^	0.008 ^c^
						Casein concentration		
						Mid-thigh fat area: ↑2 m; ↓5, 9, 12 m	NA ^e^	0.039 ^c^
						TCH concentration	NR	NS
						Lactose concentration		
						Mid-arm lean area: ↓2 m; ↑5, 9, ↓12 m	NA ^e^	0.040 ^c^
Cheema et al., 2021 Australia [37]	CS 3 m, *n* = 57 EBF to 5 m	SHM, pre-/post-feed sampling Enzymatic- spectrophotometry (Kuhn and Lowenstein method, lactose, glucose)	3 m: 793 ± 176 (512–1305) (*n* = 45) 24 h MI: pre-/post-feed test-weighing infant BC: anthropometry and BIS	(*n* = 57) Lactose, g/L 2 m: 86.56 ± 7.91 (69.91–106.09)	(*n* = 45) Lactose, g/24 h 3 m: 68.35 ± 16.63 (39.91–129.27)	Lactose concentration	NR	NS
						Lactose intake		
						↑Weight	0.017 (0.005)	0.001
						↑Length	0.047 (0.017)	0.010
						↑FFM (BIS)	0.011 (0.003)	0.001
						↑FFMI (BIS)	0.026 (0.009)	0.009
						↑FM (BIS)	0.005 (0.002)	0.011
						↑FMI (BIS)	0.014 (0.006)	0.028
						↑WAZ	0.017 (0.006)	0.007
				Glucose, g/L 2 m: 0.26 ± 0.09 (0.09–0.47)	Glucose, g/24 h 3 m: 0.20 ± 0.09 (0.05–0.49)	Glucose concentration	NR	NS
						Glucose intake		
						↑HC	4.304 (1.885)	0.028
Gridneva et al., 2021 Australia [38]	LS, 2–12 m, *n* = 20, 2 m (*n* = 15) 5 m (*n* = 20) 9 m (*n* = 19) 12 m (*n* = 18) EBF to 5 m	SHM, pre-/post-feed, morning sampling ^d^ Bradford assay (casein, total and whey protein), enzymatic spectrophotometry (Kuhn and Lowenstein method, lactose), SHM deproteinised with trichloroacetic acid, dehydrated by sulfuric acid (TCH)	5 m: 819 ± 205 (498–1185) (*n* = 17) 9 m: 478 ± 154 (300–775) (*n* = 8) 12 m: 451 ± 216 (255–795) (*n* = 8) 24 h MI: pre-/post-feed test weighing infant Regional BC: US scans of upper abdomen (preperitoneal and subcutaneous fat thickness)	Casein, whey, and total protein in Gridneva et al., 2018 (a) [42] below	Casein, whey, and total protein in Gridneva et al., 2018 (a) [42] below	Total protein concentration		
						Visceral/subcutaneous-abdominal depths ratio: ↑2, 5, 9 m; ↓12 m	NA ^e^	0.006 ^c^
						Preperitoneal fat area: ↓2 m; ↑5 m; ↓9, 12 m	NA ^e^	0.013 ^c^
						Total protein intake (overall)		
						↑Subcutaneous-abdominal fat area	0.053 (0.021)	0.013 ^b^
						Whey protein concentration and intake	NR	NS
						Casein concentration	NR	NS
						Casein intake (overall)		
						↑Subcutaneous-abdominal depth	1.34 (0.555)	0.021
				Lactose and TCH in Gridneva et al., 2019 [41] below	Lactose and TCH in Gridneva et al., 2019 [41] below	↑Subcutaneous-abdominal fat area	0.235 (0.103)	0.027
						Lactose concentration	NR	NS
						Lactose intake (overall)		
						↑Subcutaneous-abdominal depth	0.054 (0.023)	0.021
						↑Subcutaneous-abdominal fat area	0.012 (0.005)	0.013 ^b^
						TCH concentration	NR	NS
						TCH intake (overall)		
						↑Subcutaneous-abdominal depth	0.047 (0.018)	0.005
						↑Subcutaneous-abdominal fat area	0.009 (0.003)	0.004 ^b^
George et al., 2021 Australia [39]	LS, Birth–6 m (*n* = 11) EBF	WHM, pre-feed, morning and evening sampling Liquid chromatography-mass spectrometry) (targeted lipidomics analysis of 166 human milk fat globule membrane (MFGM) lipids)	3 m: 727 ± 164 (473–894) (*n* = 11) 24 h MI: pre-/post-feed test weighing infant BC: anthropometry	See paper (Supplementary Table S1) for concentrations [39]	10 lipid species with the highest mean intakes (μmol/24 h) at 3 m: PI 36:2: 17.15 ± 10.35 SM 40:1: 13.64 ± 6.51 SM 36:1: 11.05 ± 4.95 PC 36:2: 10.27 ± 7.03 SM 42:2: 9.71 ± 4.28 SM 34:1: 8.24 ± 3.92 PI 38:4: 7.21 ± 3.71 PI 36:1: 7.17 ± 4.15 PI 38:3: 7.10 ± 0.26 PC 34:1: 6.35 ± 4.26 See paper for the rest of intakes (Supplementary Table S2) and full correlation results (Supplementary Table S3) [39]	Lipid concentrations	NA	NA
						Lipids intake—strong positive correlations (*r* > 0.70) with infant anthropometry at 1, 3, and 6 m found for 99/166 MFGM lipid species		
						For 10 lipid species with the highest mean intakes:		
						PI 36:2: ↑Weight at 3 m	0.76	NA
						SM 40:1: ↑Weight at 3 m	0.68	NA
						SM 36:1: ↑Weight at 3 m	0.59	NA
						PC 36:2: ↑Weight at 3 m	0.74	NA
						SM 42:2: ↑Weight at 3 m	0.71	NA
						SM 34:1: ↑Weight at 3 m	0.65	NA
						PI 38:4: ↑Weight at 3 m	0.75	NA
						PI 36:1: ↑Weight at 3 m	0.72	NA
						PI 38:3: ↑Weight at 3 m	0.65	NA
						PC 34:1: ↑Weight at 3 m	0.72	NA
						1 m strong lipid intake correlations:	*r* > 0.80	NA
						↑HC: LPC 15:0, LPC 17:1, PC 31:0, PC-O 32:0		
						↑HCZ: SM 31:1		
						↑HC and HCZ: Cer d18:1/25:0, Cer d19:1/22:0, Cer d19:1/24:0, LPC 14:0, PC 35:2, PE 32:1, PE 35:2, PE 40:5		
						3 m strong lipid intake correlations:	*r* > 0.80	NA
						↑HC: Cer d19:1/24:0		
						6 m strong lipid intake correlations:	*r* > 0.80	NA
						↑WLZ: PI 38:5		
						↑HC: LPC 17:0, PC 31:0, PC 33:0, PC-O 32:0, PC-O 34:0, PE 35:1		
						↑HCZ: Cer d19:1/24:1, SM 31:1		
						↑HC and HCZ: Cer d18:1/23:0, Cer d18:2/23:0, Cer d19:1/22:0, Cer d19:1/24:0, LPC 14:0, LPC 15:0, LPC 16:1, LPC 17:1, PC 33:1, PC-O 34:1, SM 43:1		
	
Gridneva et al., 2019 Australia [41]	LS, 2–12 m, *n* = 20, 2 m (*n* = 15) 5 m (*n* = 20) 9 m (*n* = 19) 12 m (*n* = 18) EBF to 5 m	SHM, pre-/post-feed, morning sampling ^d^ Enzymatic- spectrophotometry (Kuhn and Lowenstein method, lactose), mid-infrared spectroscopy and UV spectrophotometry (TCH)	5 m: 819 ± 205 (498–1185) (*n* = 17) 9 m: 502 ± 158 (300–775) (*n* = 8) 12 m: 446 ± 200 (255–795) (*n* = 8) 24 h MI: re-/post-feed test weighing infant BC: anthropometry, BIS, and US skinfolds BC equations	TCH, g/L 2 m: 86.7 ± 9.2 (67.1–97.5) (*n* = 15) 5 m: 80.7 ± 7.9 (69.3–94.1) (*n* = 20) 9 m: 87.8 ± 11.1 (60.9–105.6) (*n* = 19) 12 m: 88.4 ± 21.2 (56.9–126.9) (*n* = 14)	TCH, g/24 h 2–5 m: 63.2 ± 15.0 (42.9–97.2) (*n* = 17) 9 m: 44.8 ± 15.2 (21.2–69.6) (*n* = 8) 12 m: 40.7 ± 29.8 (22.2–100.9) (*n* = 8)	TCH concentration		
						↑Length	0.047 (0.013)	<0.001 ^b^
						↑Weight	0.013 (0.004)	0.003 ^b^
						BMI ↑2 m; ↓5, 9, 12 m	NA ^e^	0.044 ^c^
						↑FFM (BIS)	0.020 (0.004)	<0.001 ^b^
						↑FFM (US2FS)	0.009 (0.004)	0.032
						↑FFM (US4FS)	0.010 (0.004)	0.020 ^b^
						↑FFMI (BIS)	0.022 (0.007)	0.002 ^b^
						FM (BIS) ↑2 m; ↓5, 9, 12 m	NA ^e^	0.016 ^b,c^
						%FM (BIS) ↑2 m; ↓5, 9, 12 m	NA ^e^	0.001 ^b,c^
						FMI (BIS) ↑2 m; ↓5, 9, 12 m	NA ^e^	0.001 ^b,c^
						FM/FFM (BIS) ↑2 m; ↓5, 9, 12 m	NA ^e^	0.003 ^b,c^
						TCH intake (overall)		
						BMI ↑2–5 m, ↓9, 12 m	NA ^e^	0.019 ^b,c^
						↓FFMI (US4SF)	–0.034 (0.007)	<0.001 ^b^
						↑FM (US2FS)	0.011 (0.004)	0.006 ^b^
						↑FM (US4FS)	0.014 (0.004)	<0.001 ^b^
						↑%FM (US2FS)	0.010 (0.036)	0.005 ^b^
						%FM (US4FS) ↑2–5 m; ↓9 m; ↑12 m	NA ^e^	0.016 ^b,c^
						↑FMI (US2FS)	0.025 (0.009)	0.003 ^b^
						FMI (US4FS) ↑2–5 m; ↓9 m; ↑12 m	NA ^e^	0.038 ^b,c^
						↑FM/FFM (US2FS)	0.002 (0.001)	0.004 ^b^
						FM/FFM (US4FS) ↑2–5 m; ↓9 m; ↑12 m	NA ^e^	0.007 ^b,c^
						TCH intake between 2 and 5 m		
						↑ΔLength 2–12 m	0.074 (0.026)	0.023
						↑Weight 2–5 m	0.018 (0.008)	0.039
						↑ΔBMI 2–5 m	0.040 (0.016)	0.037
						↑ΔFFM (BIS) 2–5 m	0.009 (0.004)	0.045
						↑ΔFFM (US4FS) 2–9 m	0.034 (0.013)	0.038
						↑ΔFFM (US4FS) 2–12 m	0.045 (0.016)	0.024
						↑ΔFM (US2FS) 2–5 m	0.024 (0.008)	0.019
						↑ΔFMI (US2FS) 2–5 m	0.050 (0.018)	0.022
						↓ΔFMI (US4FS) 9–12 m	–0.015 (0.006)	0.031
						↓Δ%FM (US4FS) 2–12 m	–0.371 (0.139)	0.032
						↓ΔFM/FFM (BIS) 2–12 m	–0.005 (0.002)	0.040
						↓ΔFM/FFM (US4FS) 2–12 m	–0.007 (0.003)	0.034
						TCH intake at 9 m		
						↓ΔBMI 5–12 m	–0.081 (0.029)	0.037
						↑ΔFFM (US2FS) 2–9 m	0.107 (0.021)	0.037
						↓ΔFM (BIS) 5–12 m	–0.033 (0.010)	0.018
						↓ΔFMI (BIS) 5–12 m	–0.077 (0.028)	0.040
						TCH intake at 12 m		
						↓ΔFFMI (US2FS) 5–12 m	–0.038 (0.014)	0.038
						↓ΔFFMI (US2FS) 9–12 m	–0.021 (0.007)	0.033
						↓ΔFFMI (US4FS) 5–12 m	0.031 (0.009)	0.029
				Lactose, g/L 2 m: 64.5 ± 4.1 (59.1–77.9) (*n* = 15) 5 m: 64.3 ± 5.9 (53.5–70.6) (*n* = 20) 9 m: 65.3 ± 5.3 (57.6–79.0) (*n* = 19) 12 m: 66.9 ± 4.0 (60.1–79.3) (*n* = 14)	Lactose, g/24 h 2–5 m: 51.2 ± 14.5 (32.6–83.6) (*n* = 17) 9 m: 34.0 ± 11.0 (19.6–51.3) (*n* = 8) 12 m: 28.7 ± 12.1 (18.0–51.4) (*n* = 8)	Lactose concentration		
						↑Length	0.065 (0.034)	0.047
						Lactose intake (overall)		
						BMI: ↑2–5, 9 m; ↓12 m	NA ^e^	0.011 ^b,c^
						FFMI (US4SF): ↑2–5, 9 m; ↓12 m	NA ^e^	0.015 ^b,c^
						↑FM (US2SF)	0.014 (0.006)	0.008 ^b^
						↑FM (US4SF)	0.015 (0.005)	0.004 ^b^
						↑%FM (US2SF	0.128 (0.050)	0.019 ^b^
						↑%FM (US4 FF)	0.156 (0.048)	0.001 ^b^
						↑FMI (BIS)	0.027 (0.013)	0.045
						↑FMI (US2SF)	0.032 (0.012)	0.005 ^b^
						↑FMI (US4SF)	0.038 (0.012)	<0.001 ^b^
						↑FM/FFM (US2SF)	0.003 (0.001)	0.012 ^b^
						↑FM/FFM (US4SF)	0.003 (0.001)	<0.001 ^b^
						Lactose intake between 2 and 5 m		
						↑ΔLength 2–12 m	0.100 (0.032)	0.016
						↑ΔBMI 2–5 m	0.049 (0.019)	0.035
						↑ΔFFM (BIS) 2–12 m	0.035 (0.012)	0.025
						↑ΔFFM (US2FS) 2–9 m	0.024 (0.009)	0.029
						↑ΔFFM (US4FS) 2–9 m	0.043 (0.012)	0.009
						↑ΔFFMI (US4FS) 2–12 m	0.088 (0.035)	0.044
						↑ΔFM (US2FS) 2–5 m	0.029 (0.010)	0.014
						↓ΔFMI (BIS) 5–12 m	–0.045 (0.017)	0.023
						↑ΔFMI (US2FS) 2–5 m	0.057 (0.023)	0.036
						↓ΔFM/FFM (BIS) 2–12 m	–0.006 (0.002)	0.032
						↓ΔFM/FFM (BIS) 5–12 m	–0.004 (0.002)	0.034
						↑ΔFM/FFM (US2FS) 2–5 m	0.007 (0.003)	0.036
						Lactose intake at 9 m		
						↓ΔFM (BIS) 5–12 m	–0.037 (0.014)	0.045
						Lactose intake at 12 m		
						↓ΔFFMI (US2FS) 5–12 m	–0.097 (0.018)	0.003
						↓ΔFFMI (US4FS) 5–12 m	–0.080 (0.007)	0.0004 ^b^
						↑ΔFM (US2FS) 2–12 m	0.036 (0.010)	0.032
Gridneva et al., 2018 (a) Australia [42]	LS, 2–12 m, *n* = 20, 2 m (*n* = 13) 5 m (*n* = 20) 9 m (*n* = 18) 12 m (*n* = 13) EBF to 5 m	SHM, pre-/post-feed, morning sampling ^d^ Kunz and Lonnerdal (casein and whey protein fraction separation) Bradford assay (protein)	5 m: 818.8 ± 204.9 (498–1185) (*n* = 17) 9 m: 478 ± 154 (300–775) (*n* = 8) 12 m: 451.1 ± 215.7 (255–795) (*n* = 8) 24 h MI: pre-/post-feed test weighing infant BC: anthropometry, BIS, and US skinfolds BC equations	Total protein, g/L 2 m: 11.03 ± 1.40 (7.60–12.32) (*n* = 15) 5 m: 11.90 ± 4.31 (7.93–24.16) (*n* = 20) 9 m: 9.69 ± 1.12 (7.25–14.96) (*n* = 19) 12 m: 10.72 ± 2.84 (5.89–16.80) (*n* = 15)	Total protein, g/24 h 2–5 m: 9.19 ± 3.82 (4.51–20.34) (*n* = 17) 9 m: 5.24 ± 1.84 (2.18–7.48) (*n* = 8) 12 m: 4.18 ± 2.11 (1.93–7.23) (*n* = 8)	Total protein concentration		
						↓FFMI (BIS)	–0.034 (0.022)	0.032
						Total protein intake (overall)	NR	NS
						Total protein intake between 2 and 5 m		
						↑ΔHC 9–12 m	0.09 (0.04)	0.046
						↑ΔLength 2–9 m	0.40 (0.15)	0.025
						↑ΔLength 2–12 m	0.57 (0.17)	0.011
						↑ΔBMI 2–5 m	0.29 (0.09)	0.013
						↑ΔFFM (BIS) 2–12 m	0.18 (0.07)	0.041
						↑ΔFFM (BIS) 9–12 m	0.08 (0.03)	0.041
						↑ΔFFM (US4FS) 2–9 m	0.19 (0.07)	0.037
						↑ΔFFMI (BIS) 2–5 m	0.14 (0.06)	0.042
						↑ΔFFMI (US4FS) 2–5 m	0.27 (0.09)	0.023
						↑ΔFM (US2FS) 2–5 m	0.13 (0.06)	0.049
						↑ΔFMI (BIS) 2–5 m	0.17 (0.07)	0.044
						↓ΔFMI (US2FS) 9–12 m	–0.07 (0.03)	0.029
						Total protein intake at 9 m		
						↓ΔHC 9–12 m	–0.30 (0.09)	0.021
						↓ΔFFMI (BIS) 2–5 m	–0.36 (0.02)	0.004
						Total protein intake at 12 m		
						↓ΔBMI 9–12 m	–0.30 (0.09)	0.021
						↓ΔFFMI (US2FS) 5–12 m	–0.48 (0.18)	0.045
						↓ΔFFMI (US4FS) 5–12 m	–0.50 (0.09)	0.006
						↑ΔFM (US2FS) 2–12 m	0.21 (0.05)	0.024
						↑ΔFM (US2FS) 5–12 m	0.14 (0.06)	0.043
						↑Δ%FM (US2SF) 5–12 m	1.60 (0.59)	0.036
						↑ΔFMI (US2FS) 2–12 m	0.38 (0.08)	0.045
				Whey protein, g/L 2 m: 6.44 ± 1.62 (4.12–9.08) (*n* = 15) 5 m: 5.43 ± 0.90 (3.82–7.38) (*n* = 20) 9 m: 5.43 ± 0.93 (3.94–9.40) (*n* = 19) 12 m: 7.61 ± 1.85 (4.49–9.76) (*n* = 15)	Whey protein, g/24 h 2–5 m: 4.23 ± 1.14 (2.65–6.76) (*n* = 17) 9 m: 3.0 2 ±1.11 (1.70–4.64) (*n* = 8) 12 m: 2.78 ± 1.34 (1.15–4.40) (*n* = 8)	Whey protein concentration	NR	NS
						Whey protein intake (overall)		
						↓FFMI (BIS)	–0.319 (0.121)	0.004
						↑FM (US2SF)	0.148 (0.062)	0.024
						↑%FM (US2SF)	1.330 (0.554)	0.033
						↑%FM (U42 SF)	1.190 (0.565)	0.038
						↑FMI (US2SF)	0.336 (0.135)	0.016
						↑FMI (US4SF)	0.309 (0.143)	0.034
						Whey protein intake between 2 and 5 m		
						↑ΔLength 2–12 m	1.01 (0.35)	0.024
						↑ΔBMI 2–5 m	0.58 (0.19)	0.017
						↑ΔFFMI (US4FS) 2–5 m	0.65 (0.21)	0.016
						↑ΔFM (US2FS) 2–5 m	0.29 (0.12)	0.032
						↑ΔFMI (US2FS) 2–5 m	0.61 (0.25)	0.042
						Whey protein intake at 9 m	NR	NS
						Whey protein intake at 12 m		
						↓ΔFFMI (US4FS) 5–12 m	–0.62 (0.22)	0.049
						↑ΔFM (US2SF) 2–12 m	0.26 (0.06)	0.025
				Casein, g/L 2 m: 1.24 ± 0.24 (0.69–1.57) (*n* = 15) 5 m: 1.51 ± 0.44 (0.78–3.45) (*n* = 20) 9 m: 1.11 ± 0.38 (0.49–2.00) (*n* = 19) 12 m: 1.07 ± 0.35 (0.65–1.87) (*n* = 15)	Casein, g/24 h 2–5 m: 1.45 ± 0.82 (0.56–3.63) (*n* = 17) 9 m: 0.60 ± 0.23 (0.17–0.95) (*n* = 8) 12 m: 0.54 ± 0.34 (0.24–1.19) (*n* = 8)	Casein–whey ratio		
						↑%FM (US4 FF)	9.09 (4.62)	0.046
						Casein concentration	NR	NS
						Casein intake (overall)		
						HC: ↓2–5 m; ↑9 m; ↓12 m	NA ^e^	0.046 ^c^
						BMI: ↑2–5 m; ↓9, 12 m	NA ^e^	0.047 ^c^
						↓FFM (US4SF)	–0.456 (0.144)	0.003 ^b^
						↓FFMI (US4SF) ↓2–5, 9, 12 m	NA ^e^	0.022 ^b,c^
						↑FM (US4SF)	0.390 (0.100)	<0.001 ^b^
						↑%FM (US4SF)	4.220 (0.932)	<0.001 ^b^
						↑FMI (US2SF)	0.517 (0.248)	0.048
						↑FMI (US4SF)	0.789 (0.240)	0.001 ^b^
						Casein intake between 2 and 5 m		
						↓ΔHC 2–5 m	–1.17 (0.39)	0.016
						↓ΔFM (US4FS) 2–9 m	–0.47 (0.17)	0.031
						↓ΔFM (US4FS) 2–12 m	–0.49 (0.20)	0.040
						↓Δ%FM (BIS) 2–12 m	–4.48 (1.61)	0.027
						↓Δ%FM (US2FS) 2–9 m	–3.62 (1.50)	0.042
						↓Δ%FM (US4FS) 2–9 m	–6.67 (1.36)	0.002
						↓Δ%FM (US4FS) 2–12 m	–6.27 (2.42)	0.036
						↓ΔFMI (US4FS) 2–9 m	–1.29 (0.32)	0.005
						↓ΔFMI (US4FS) 2–12 m	–1.44 (0.50)	0.028
						Casein intake at 9 m		
						↓ΔHC 5–12 m	–1.78 (0.62)	0.036
						↓ΔHC 9–12 m	–2.40 (0.74)	0.023
						Casein intake at 12 m		
						↑ΔHC 2–12 m	4.01 (0.60)	0.022
						↓ΔBMI between 9 and 12 m	–2.09 (0.61)	0.019
						↓ΔFFM (US4FS) 2–12 m	–3.77 (0.73)	0.014
						↓ΔFFMI (US2FS) 9–12 m	–2.13 (0.45)	0.005
						↓ΔFFMI (US4FS) 5–12 m	–2.84 (0.87)	0.031
						↓ΔFFMI (US4FS) 9–12 m	–2.40 (0.78)	0.028
						↑Δ%FM (US4FS) 2–12 m	33.56 (8.59)	0.030
Kon et al., 2014 Russian Federation [44]	CS, 1, 2 or 3 m, *n* = 103, 1 m (*n* = 32) 2 m (*n* = 34) 3 m (*n* = 33) LWG (<500 g/m, *n* = 18) NWG (500–1000 g/m, *n* = 40) HWG (>1000 g/m, *n* = 45) EBF	SHM, midstream morning sampling Van de Kamer method (fat), Kjeldahl and Lowry technique (protein)	Combined mean (calculated): 1 m: 736 (*n* = 32) 2 m: 826 (*n* = 34; no LWG) 3 m: 891 (*n* = 33) LWG 1 m: 555 ± 32 (*n* = 9) 2 m: NA 3 m: 753 ± 53 (*n* = 5) NWG 1 m: 849 ± 96 (*n* = 11) 2 m: 752 ± 93 (*n* = 14) 3 m: 896 ± 42 (*n* = 15) HWG 1 m: 768 ± 41 (*n* = 12) 2 m: 878 ± 37 (*n* = 20) 3 m: 937 ± 70 (*n* = 13) 24 h MI: pre-/post-feed test weighing infant BC: weight gain	Fat, g/100 g LWG 1 m: 4.08 ± 0.45 (*n* = 9) 2 m: NA (*n* = 4, excluded) 3 m: 2.51 ± 0.26 (*n* = 5) NWG 1 m: 4.39 ± 0.84 (*n* = 11) 2 m: 4.43 ± 0.52 (*n* = 14) 3 m: 3.76 ± 0.47 (*n* = 15) HWG 1 m: 4.51 ± 0.57 (*n* = 12) 2 m: 3.65 ± 0.36 (*n* = 20) 3 m: 3.80 ± 0.25 (*n* = 13) Protein g/100 g LWG 1 m: 1.77 ± 0.22 (*n* = 9) 2 m: NA (*n* = 4, excluded) 3 m: 1.50 ± 0.09 (*n* = 5) NWG 1 m: 1.70 ± 0.10 (*n* = 11) 2 m: 1.68 ± 0.14 (*n* = 14) 3 m: 1.32 ± 0.08 (*n* = 15) HWG 1 m: 1.66 ± 0.16 (*n* = 12) 2 m: 1.77 ± 0.12 (*n* = 20) 3 m: 1.50 ± 0.20 (*n* = 13)	Fat, g/24 h LWG 1 m: 24.6 ± 4.4 (*n* = 9) 2 m: NA (*n* = 4, excluded) 3 m: 19.1 ± 2.8 (*n* = 5) NWG 1 m: 34.4 ± 3.6 (*n* = 11) 2 m: 34.7 ± 6.5 (*n* = 14) 3 m: 35.3 ± 4.4 (*n* = 15) HWG 1 m: 38.6 ± 6.6 (*n* = 12) 2 m: 31.3 ± 2.9 (*n* = 20) 3 m: 36.2 ± 2.5 (*n* = 13) Protein, g/24 h LWG 1 m: 10.2 ± 1.4 (*n* = 9) 2 m: NA (*n* = 4, excluded) 3 m: 11.3 ± 1.2 (*n* = 5) NWG 1 m: 15.2 ± 2.4 (*n* = 11) 2 m: 12.1 ± 1.8 (*n* = 14) 3 m: 11.7 ± 1.1 (*n* = 15) HWG 1 m: 12.2 ± 1.1 (*n* = 12) 2 m: 15.2 ± 1.2 (*n* = 20) 3 m: 14.1 ± 2.7 (*n* = 13)	Fat concentration		
						No significant difference between LWG, NWG, and HWG groups at any time point	Concentrations by group and time point are on the left	NS
		
						Fat intake		
						No significant difference between LWG, NWG, and HWG groups at 2 and 3 m	Intakes by group and time point are on the left	NS
						↑Fat intake at 3 m in the HWG group compared with the LWG group		<0.05
		
						Protein concentration		
						No significant difference between LWG, NWG, and HWG groups at any time point	Concentrations by group and time point are on the left	NS
						Protein intake		
						No significant difference between LWG, NWG, and HWG groups at any time point	Intakes by group and time point are on the left	NS
		

Mitoulas et al., 2002 Australia [29]	LS, 1–12 m, *n* = 17, 1 m (*n* = 17) 2 m (*n* = 17) 4 m (*n* = 17) 6 m (*n* = 15) 9 m (*n* = 6) 12 m (*n* = 5) EBF to 4 m	WHM (fat), SHM (lactose and protein), pre-/post-feed sampling Colorimetric spectrophotometry Stern and Shapiro (fat), enzymatic-spectrophotometry (Kuhn and Lowenstein method, lactose), Bradford assay (protein)	24 h MI averaged by the breast: 1 m: 416 ± 24 (*n* = 34) 2 m: 408 ± 23 (*n* = 34) 4 m: 421 ± 20 (*n* = 34) 6 m: 413 ± 25 (*n* = 30) 9 m: 354 ± 47 (*n* = 12) 12 m: 252 ± 51 (*n* = 10) 1–12 m: 399 ± 11 (*n* = 154) 24 h MI: pre-/post-feed test weighing the mother, corrected for insensible water loss BC: anthropometry	Fat, g/L 1 m: 39.9 ± 1.4 (*n* = 17) 2 m: 35.2 ± 1.4 (*n* = 17) 4 m: 35.4 ± 1.4 (*n* = 16) 6 m: 37.3 ± 1.4 (*n* = 14) 9 m: 40.7 ± 1.7 (*n* = 6) 12 m: 40.9 ± 3.3 (*n* = 5) Lactose, g/L 1 m: 59.7 ± 0.8 (*n* = 9) 2 m: 60.4 ± 1.1 (*n* = 9) 4 m: 62.6 ± 1.3 (*n* = 8) 6 m: 62.5 ± 1.7 (*n* = 8) 9 m: 62.8 ± 1.5 (*n* = 6) 12 m: 61.4 ± 2.9 (*n* = 5) Protein, g/L 1 m: 10.5 ± 0.4 (*n* = 9) 2 m: 9.6 ± 0.37 (*n* = 9) 4 m: 9.33 ± 0.42 (*n* = 9) 6 m: 8.03 ± 0.38 (*n* = 8) 9 m: 8.34 ± 0.45 (*n* = 6) 12 m: 8.34 ± 0.57 (*n* = 5)	Intake per breast Fat, g/24 h 1 m: 16.4 ± 1.2 (*n* = 17) 2 m: 14.2 ± 0.95 (*n* = 17) 4 m: 14.3 ± 0.6 (*n* = 16) 6 m: 15.7 ± 0.9 (*n* = 14) 9 m: 14.3 ± 1.9 (*n* = 6) 12 m: 10.4 ± 2.0 (*n* = 5) Lactose, g/24 h 1 m: 22.9 ± 2.0 (*n* = 9) 2 m: 25.4 ± 2.2 (*n* = 9) 4 m: 27.0 ± 2.2 (*n* = 8) 6 m: 25.1 ± 2.1 (*n* = 8) 9 m: 22.4 ± 3.2 (*n* = 6) 12 m: 17.4 ± 3.5 (*n* = 5) Protein, g/24 h 1 m: 4.0 ± 0.4 (*n* = 9) 2 m: 4.05 ± 0.41 (*n* = 9) 4 m: 3.83 ± 0.23 (*n* = 9) 6 m: 3.13 ± 0.2 (*n* = 8) 9 m: 2.94 ± 0.39 (*n* = 6) 12 m: 2.14 ± 0.41 (*n* = 5)	Fat concentration and intake		
						1–6 m Weight	NR	NS
						1–6 m Weight gain	NR	NS
		
		
						Lactose concentration and intake		
						1–6 m Weight	NR	NS
						1–6 m Weight gain	NR	NS
		
		
		
		
		
						Protein concentration and intake 1–6 m Weight 1–6 m Weight gain	NR NR	NS NS
		
		
		
		
		
		
Heinig et al., 1993 United States of America [45]	LS, 3–18 m, *n* = 60, 3 m (*n* = 71) 6 m (*n* = 56) 9 m (*n* = 46) 12 m (*n* = 40) 15 m (*n* = NR) 18 m (*n* = NR) EBF to 4 m	WHM, 24 h alternate breast expression Kjeldahl and Lowry methods (protein), modified Folch extraction (lipid), Dahlquist lactase assay (lactose)	24 h MI, uncorrected: 3 m: 768 ± 127 6 m: 732 ± 165 9 m: 611 ± 211 12 m: 413 ± 246 Corrected for insensible water loss: 3 m: 812 ± 133 6 m: 769 ± 171 9 m: 646 ± 217 12 m: 448 ± 251 24 h MI: pre-/post-feed test-weighing infant and 4 d weighed food record BC: total body water (isotope-dilution method) and anthropometry	Protein, g/24 h 3 m: 8.7 6 m: 8.1 9 m: 8.4 12 m: 10.0 Fat concentration, stable and averaged throughout 3–12 m: 37 g/L Lactose concentration, stable and averaged throughout 3–12 m: 74 g/L	Protein, g/24 h 3 m: 6.8 ± 1.1 (*n* = 71) 6 m: 5.9 ± 1.3 (*n* = 56) 9 m: 5.0 ± 1.6 (*n* = 46) 12 m: 3.8 ± 1.9 (*n* = 40) Protein intake from human milk % 3 m: 100% 6 m: 78 ± 20% 9 m: 46 ± 25% 12 m: 22 ± 17% Lactose and fat intakes NR, only used for calculation of energy intake	No concentrations and BC analysis	NR	NR
						Total protein intake		
						↑Weight gain 3–6 m	0.30	<0.01
						↑Lean body mass gain 3–6 m	0.34	<0.01
						↑Weight gain 6–9 m	0.34	<0.01
						↑Lean body mass gain 6–9 m	0.34	<0.05
						↑Lean body mass gain 12–15 m	0.33	<0.05

^a^ Data are mean ± SD, median [IQR], mean difference (95% CI), and/or β (parameter estimate) (SEE); only results significant prior to and post multiple comparison adjustment are presented in detail. ^b^ The results are significant after multiple comparisons. ^c^ *p*-value reported is for significant interaction. ^d^ Sampling time reported by the author on request. ^e^ Overall ß (SE) are not available when a significant interaction with age is present; individual ß (SE) reported for 2, 5, 9, and 12 months. BC, body composition; BIS, bioelectrical impedance spectroscopy; BMI, body mass index; CS, cross-sectional study; d, day; EBF, exclusively breastfed; FFM, fat-free mass; FFMI, fat-free mass index; FM, fat mass; FMI, fat mass index; HC, head circumference; HCZ, head circumference z-score; HWG, high weight gain; LAZ, length-for-age z-score; LS, longitudinal study; LWG, low weight gain; m, month; MI, milk intake; MFGM, milk fat globule membrane; NA, not available; NMR, ^1^ H-nuclear magnetic resonance; NR, not reported; NS, not significant; NW, normal weight; NWG, normal weight gain; OW, overweight; SDS, standard deviation scores; SEE, standard error of estimate; SHM, skim human milk; TCH, total carbohydrates; US, ultrasound; US2SF, ultrasound 2-skinfolds; US4SF, ultrasound 4-skinfolds; w, week; WAZ, weight-for-age z-score; WLZ, weight-for-length z-score; WHM, whole human milk. ↓, lower; ↑, higher.

**Table A2:** Summary of intakes of human milk macronutrients presented by time postpartum.

Human Milk Macronutrient	Time Postpartum	Sample Size	Macronutrient Intake, g/24 h	Comments	Author, Year
Total protein	1 m	*n* = 9 *n* = 11 *n* = 12	10.2 ± 1.4 ^a^ 15.2 ± 2.4 12.2 ± 1.1	LWG NWG HWG	Kon et al., 2014 [44]
		*n* = 9	4.0 ± 0.4	Intake per breast	Mitoulas et al., 2002 [29]
	6 w	*n* = 47	9.1 ± 2.1		Olga et al., 2022 [34]
	2 m	*n* = 4 *n* = 14 *n* = 20	NA 12.1 ± 1.8 15.2 ± 1.2	LWG NWG HWG	Kon et al., 2014 [44]
		*n* = 9	4.05 ± 0.41	Intake per breast	Mitoulas et al., 2002 [29]
	3 m	*n* = 5 *n* = 15 *n* = 13	11.3 ± 1.2 11.7 ± 1.1 14.1 ± 2.7	LWG NWG HWG	Kon et al., 2014 [44]
		*n* = 71	6.8 ± 1.1	Protein intake from human milk at 3 m: 100%	Heinig et al., 1993 [45]
	2–5 m	*n* = 17	9.19 ± 3.82 (4.51–20.34)		Gridneva et al., 2018 (a) [42]
	4 m	*n* = 9	3.83 ± 0.23	Intake per breast	Mitoulas et al., 2002 [29]
	6 m	*n* = 8	3.13 ± 0.2	Intake per breast	Mitoulas et al., 2002 [29]
		*n* = 56	5.9 ± 1.3	Protein intake from human milk at 6 m: 78 ± 20%	Heinig et al., 1993 [45]
	9 m	*n* = 8	5.24 ± 1.84 (2.18–7.48)		Gridneva et al., 2018 (a) [42]
		*n* = 6	2.94 ± 0.39	Intake per breast	Mitoulas et al., 2002 [29]
		*n* = 46	5.0 ± 1.6	Protein intake from human milk at 9 m: 46 ± 25%	Heinig et al., 1993 [45]
	12 m	*n* = 8	4.18 ± 2.11 (1.93–7.23)		Gridneva et al., 2018 (a) [42]
		*n* = 5	2.14 ± 0.41	Intake per breast	Mitoulas et al., 2002 [29]
		*n* = 40	3.8 ± 1.9	Protein intake from human milk at 12 m: 22 ± 17%	Heinig et al., 1993 [45]
Casein	2–5 m	*n* = 17	1.45 ± 0.82 (0.56–3.63)		Gridneva et al., 2018 (a) [42]
	9 m	*n* = 8	0.60 ± 0.23 (0.17–0.95)		Gridneva et al., 2018 (a) [42]
	12 m	*n* = 8	0.54 ± 0.34 (0.24–1.19)		Gridneva et al., 2018 (a) [42]
Whey protein	2–5 m	*n* = 17	4.23 ± 1.14 (2.65–6.76)		Gridneva et al., 2018 (a) [42]
	9 m	*n* = 8	3.0 2 ± 1.11 (1.70–4.64)		Gridneva et al., 2018 (a) [42]
	12 m	*n* = 8	2.78 ± 1.34 (1.15–4.40)		Gridneva et al., 2018 (a) [42]
Total carbohydrates	2–5 m	*n* = 17	63.2 ± 15.0 (42.9–97.2)		Gridneva et al., 2019 [41]
	9 m	*n* = 8	44.8 ± 15.2 (21.2–69.6)		Gridneva et al., 2019 [41]
	12 m	*n* = 8	40.7 ± 29.8 (22.2–100.9)		Gridneva et al., 2019 [41]
Lactose	1 m	*n* = 9	22.9 ± 2.0	Intake per breast	Mitoulas et al., 2002 [29]
	6 w	*n* = 47	50.8 ± 11.7		Olga et al., 2022 [34]
	2 m	*n* = 9	25.4 ± 2.2	Intake per breast	Mitoulas et al., 2002 [29]
	3 m	*n* = 45	68.35 ± 16.63 (39.91–129.27)		Cheema et al., 2021 [37]
	2–5 m	*n* = 17	51.2 ± 14.5 (32.6–83.6)		Gridneva et al., 2019 [41]
	4 m	*n* = 8	27.0 ± 2.2	Intake per breast	Mitoulas et al., 2002 [29]
	6 m	*n* = 8	25.1 ± 2.1	Intake per breast	Mitoulas et al., 2002 [29]
	9 m	*n* = 8	34.0 ± 11.0 (19.6–51.3)		Gridneva et al., 2019 [41]
		*n* = 6	22.4 ± 3.2	Intake per breast	Mitoulas et al., 2002 [29]
	12 m	*n* = 8	28.7 ± 12.1 (18.0–51.4)		Gridneva et al., 2019 [41]
		*n* = 5	17.4 ± 3.5	Intake per breast	Mitoulas et al., 2002 [29]
Glucose	3 m	*n* = 45	0.20 ± 0.09 (0.05–0.49)		Cheema et al., 2021 [37]
Fat	1 m	*n* = 9 *n* = 11 *n* = 12	24.6 ± 4.4 34.4 ± 3.6 38.6 ± 6.6	LWG NWG HWG	Kon et al., 2014 [44]
		*n* = 17	16.4 ± 1.2	Intake per breast	Mitoulas et al., 2002 [29]
	6 w	*n* = 47	29.6 ± 17.4		Olga et al., 2022 [34]
	2 m	*n* = 4 *n* = 14 *n* = 20	NA 34.7 ± 6.5 31.3 ± 2.9	LWG NWG HWG	Kon et al., 2014 [44]
		*n* = 17	14.2 ± 0.95	Intake per breast	Mitoulas et al., 2002 [29]
	3 m	*n* = 5 *n* = 15 *n* = 13	19.1 ± 2.8 35.3 ± 4.4 36.2 ± 2.5	LWG NWG HWG	Kon et al., 2014 [44]
	4 m	*n* = 16	14.3 ± 0.6	Intake per breast	Mitoulas et al., 2002 [29]
	6 m	*n* = 14	15.7 ± 0.9	Intake per breast	Mitoulas et al., 2002 [29]
	9 m	*n* = 6	14.3 ± 1.9	Intake per breast	Mitoulas et al., 2002 [29]
	12 m	*n* = 5	10.4 ± 2.0	Intake per breast	Mitoulas et al., 2002 [29]
MFGM lipids	1 m 3 m	*n* = 11 *n* = 11	See paper for intakes of 166 lipids at 1 m and 3 m [39]		George et al., 2021 [39]

^a^ Data are mean ± SD (standard deviation) and min–max where available. HWG, high weight gain; LWG, low weight gain; m, month; MFGM, milk fat globule membrane; NA, not available; NWG, normal weight gain; w, week.

**Table A3:** Summary of studies investigating the relationships between intakes of human milk bioactive molecules and infant anthropometrics and body composition.

Author, Year, Country	Study Type, Time Postpartum, Sample Size	Sample Type, Collection, Analyses	24 h Milk Intake and Body Composition Methods	Component Concentration	Component 24 h Intake	Association with Anthropometry or Body Composition	ß (SE) or *r*	*p*-Value
Gridneva et al., 2022 Australia [35]	LS, 2–12 m, *n* = 20, 2 m (*n* = 14) 5 m (*n* = 20) 9 m (*n* = 18) 12 m (*n* = 18) EBF to 5 m	SHM (WHM for adiponectin and leptin), pre-/post-feed, morning sampling ^d^ Enzymatic spectrophotometry (lactose); deproteinisation with trichloroacetic acid, dehydration by sulfuric acid (TCH) for calculation of total HMO concentration, Selsted and Martinez method (lysozyme), ELISA (adiponectin, whole milk leptin, lactoferrin, sIgA)	5 m: 819 ± 205 (498–1185) (*n* = 17) 9 m: 478 ± 154 (300–775) (*n* = 8) 12 m: 451 ± 216 (255–795) (*n* = 8) 24 h MI: pre-/post-feed test weighing infant Regional BC: US scans of anterior upper arm and thigh	Adiponectin and whole milk leptin in Gridneva et al., 2018 (b) [43] below Total HMOs in Gridneva et al., 2019 [41] below Lactoferrin, lysozyme and sIgA in Gridneva et al., 2020 [40] below	Adiponectin and whole milk leptin in Gridneva et al., 2018 (b) [43] below Total HMOs in Gridneva et al., 2019 [41] below Lactoferrin, lysozyme and sIgA in Gridneva et al., 2020 [40] below	Adiponectin concentration		
						↓Mid-thigh fat area	NA ^e^	0.028 ^c^
						Adiponectin intake (overall)	NR	NS
						Whole milk leptin concentration	NR	NS
						Whole milk leptin intake (overall)		
						Mid-arm lean area: ↑2 m; ↓5 m; ↑9 m; ↓12 m	NA ^e^	0.046 ^c^
						↓Mid-thigh lean area	−0.06 (0.03)	0.025
						Total HMO concentration	NR	NS
						Total HMO intake (overall)		
						Mid-arm fat area: ↓2 m; ↑5, 9, 12 m	NA ^e^	0.004 ^b,c^
						Lactoferrin concentration		
						Mid-arm lean area: ↓2, 5 m; ↑9, 12 m	NA ^e^	0.029 ^c^
						Mid-thigh lean area: ↓2, 5 m; ↑9 m; ↓12 m	NA ^e^	0.040 ^c^
						Lactoferrin intake (overall)		
						sIgA concentration	NR	NS
						Mid-arm lean area: ↓2 m; ↑5, 9, 12 m	NA ^e^	0.007 ^c^
						Mid-arm fat area: ↑2, 5 m; ↓9, 12 m	NA ^e^	0.041 ^c^
						sIgA intake (overall)	NR	NS
						Lysozyme concentration	NR	NS
						Lysozyme intake (overall)		
						Mid-arm fat area: ↑2, 5, 9 m; ↓12 m	NA ^e^	0.001 ^b,c^
Cheema et al., 2022 Australia [36]	CS, 3 m, *n* = 60 EBF to 5 m Maternal secretor status: secretor (*n* = 49), non-secretor (*n* = 11)	WHM, pre-/post-feed sampling HPLC (19 individual HMOs: 2′-fucosyllactose (2′FL), 3′-sialyllactose (3′SL), 3-fucosyllactose (3 FL), 6′-sialyllactose (6′SL), difucosyllactose (DFLac), difucosyllacto-*n*-hexaose (DFLNH), difucosyllacto-*n*-tetrose (DFLNT), disialyllacto-*n*-hexaose (DSLNH), disialyllacto-*n*-tetraose (DSLNT), fucodisialyllacto-*n*-hexaose (FDSLNH), fucosyllacto-*n*-hexaose (FLNH), lacto-*n*-fucopentaose (LNFP I), lacto-*n*-fucopentaose II (LNFP II), lacto-*n*-fucopentaose III (LNFP III), lacto-*n*-hexaose (LNH), lacto-*n*-neotetraose (LNnT), lacto-*n*-tetrose (LNT), sialyl-lacto-*n*-tetraose b (LSTb), sialyl-lacto-*n*-tetraose c (LSTc)	3 m: 785 ± 172 (512–1305) (*n* = 47) 24 h MI: pre-/post-feed test-weighing infant BC: anthropometry and BIS	HMOs, μg/mL Individual HMO concentrations in entire cohort and by maternal secretor status in Cheema et al., 2022 [36]	HMOs, μg/24 h Individual HMO intakes in entire cohort and by maternal secretor status in Cheema et al., 2022 [36]	For results by maternal secretor status, see Cheema et al., 2022 [36]; in entire cohort:		
						2′FL concentration	NR	NS
						2′FL intake		
						↑Weight	0.00 (0.00)	0.03
						↑FM	0.02 (0.01)	0.04
						3 FL concentration	NR	NS
						3 FL intake		
						↑Weight	0.09 (0.03)	0.01
						↑Length	0.20 (0.08)	0.01
						↑WAZ	0.10 (0.04)	0.01
						↑LAZ	0.08 (0.04)	0.04
						↑Log FFM	0.01 (00.0)	0.01
						↑Log FFMI	0.01 (00.0)	0.04
						Log (DFLac) concentration	NR	NS
						Log (DFLac) intake		
						↑Weight	0.33 (0.14)	0.02
						↑BMI	0.51 (0.23)	0.03
						↑BMIAZ	0.28 (0.13)	0.04
						↑Log FFM	0.05 (0.02)	0.02
						↑Log FFMI	0.04 (0.01)	0.02
						Log (DFLNH) concentration		
						↑ Weight	0.24 (0.11)	0.03
						↑Length	1.1 (0.36)	<0.001
						↑LAZ	0.44 (0.17)	0.01
						↑Log FFM	0.04 (0.02)	0.02
						Log (DFLNH) intake		
						↑Weight	0.28 (0.11)	0.01
						↑Length	1.22 (0.35)	<0.001
						↑WAZ	0.28 (0.13)	0.03
						↑LAZ	0.48 (0.17)	0.01
						↑Log FFM	0.04 (0.02)	0.01
						DFLNT concentration	NR	NS
						DFLNT intake		
						↑BMI	0.12 (0.05)	0.04
						↑ BMIAZ	0.07 (0.03)	0.03
						↑Log FFMI	0.01 (0.00)	0.03
						FLNH concentration		
						↓Weight	–0.03 (0.01)	0.04
						FLNH intake	NR	NS
						Log (LNFP III) concentration		
						↓%FM	–1.6 (0.64)	0.02
						↓FM/FFM	–0.03 (0.01)	0.02
						Log (LNFP III) intake	NR	NS
						Log (LNnT) concentration		
						↓Length	–1.11 (0.5)	0.03
						↓LAZ	–0.51 (0.23)	0.03
						Log (LNnT) intake	NR	NS
						LSTb concentration	NR	NS
						LSTb intake		
						↑BMI	0.83 (0.39)	0.04
Cheema et al., 2021 Australia [37]	CS, 3 m, *n* = 57 EBF to 5 m	WHM, pre-/post-feed sampling ELISA (insulin)	3 m: 793 ± 176 (512–1305) (*n* = 45) 24 h MI: pre-/post-feed test-weighing infant BC: anthropometry and BIS	Insulin, ng/mL 2 m: 1.48 ± 1.14 (0.46–5.90) (*n* = 57)	Insulin, ng/24 h 3 m: 1.16 ± 0.78 (0.30–4.17) (*n* = 45)	Insulin concentration	NR	NS
						Insulin intake	NR	NS
Gridneva et al., 2021 Australia [38]	LS, 2–12 m, *n* = 20, 2 m (*n* = 15) 5 m (*n* = 20) 9 m (*n* = 19) 12 m (*n* = 18) EBF to 5 m	SHM (WHM for adiponectin and leptin), pre-/post-feed, morning sampling ^d^ Enzymatic spectrophotometry (lactose); deproteinisation with trichloroacetic acid, dehydration by sulfuric acid (TCH) for calculation of total HMO concentration, Selsted and Martinez method (lysozyme), ELISA (adiponectin, whole milk leptin, lactoferrin, sIgA)	5 m: 819 ± 205 (498–1185) (*n* = 17) 9 m: 478 ± 154 (300–775) (*n* = 8) 12 m: 451 ± 216 (255–795) (*n* = 8) 24 h MI: pre-/post-feed test-weighing infant Regional BC: US scans of upper abdomen (preperitoneal and subcutaneous fat thickness)	Adiponectin and whole milk leptin in Gridneva et al., 2018 (b) [43] below Total HMOs in Gridneva et al., 2019 [41] below Lactoferrin, lysozyme, and sIgA in Gridneva et al., 2020 [40] below	Adiponectin and whole milk leptin in Gridneva et al., 2018 (b) [43] below Total HMOs in Gridneva et al., 2019 [41] below Lactoferrin, lysozyme, and sIgA in Gridneva et al., 2020 [40] below	Adiponectin concentration		
						Subcutaneous-abdominal depth: ↓2 m; ↑5 m; ↓9, 12 m	NA ^e^	0.041 ^c^
						Adiponectin intake (overall)		
						Subcutaneous-abdominal depth: ↓2 m; ↑5 m; ↓9, 12 m	NA ^e^	0.027 ^c^
						Whole milk leptin concentration	NR	NS
						Whole milk leptin intake (overall)	NR	NS
						Total HMO concentration	NR	NS
						Total HMO intake (overall)	NR	NS
						Lactoferrin concentration		
						Visceral depth: ↓2 m; ↑5, 9 m; ↓12 m	NA ^e^	0.003 ^b,c^
						Lactoferrin intake (overall)		
						Preperitoneal/subcutaneous-abdominal fat areas ratio: ↓2 m; ↑5 m; ↓9 m; ↑12 m	NA ^e^	0.028 ^c^
						Lysozyme concentration		
						↓Visceral depth	–32.5 (14.1)	0.019
						Lysozyme intake (overall)	NR	NS
						sIgA concentration		
						Preperitoneal fat area: ↓2 m; ↑5 m; ↓9, 12 m	NA ^e^	0.049 ^c^
						sIgA intake (overall)		
						Subcutaneous-abdominal depth: ↓2 m; ↑5 m; ↓9, 12 m	NA ^e^	0.029 ^c^
						↑Subcutaneous-abdominal fat area	0.742 (0.379)	0.042
Gridneva et al., 2020 Australia [40]	LS, 2–12 m, *n* = 20, 2 m (*n* = 14) 5 m (*n* = 20) 9 m (*n* = 18) 12 m (*n* = 18) EBF to 5 m	SHM, pre-/post-feed, morning sampling ^d^ Selsted and Martinez method (lysozyme), ELISA (lactoferrin and sIgA)	5 m: 819 ± 205 (498–1185) (*n* = 17) 9 m: 502 ± 158 (300–775) (*n* = 8) 12 m: 446 ± 200 (255–795) (*n* = 8) 24 h MI: pre-/post-feed test-weighing infant BC: anthropometry, BIS, and US skinfolds BC equations	Lactoferrin, g/L 2 m: 0.44 ± 0.22 (0.03–1.04) (*n* = 14) 5 m: 0.41 ± 0.17 (0.07–0.75) (*n* = 20) 9 m: 0.59 ± 0.25 (0.29–1.16) (*n* = 19) 12 m: 0.63 ± 0.20 (0.30–1.02) (*n* = 15)	Lactoferrin, g/24 h 2–5 m: 0.29 ± 0.12 (0.06–0.51) (*n* = 17) 9 m: 0.31 ± 0.21 (0.13–0.74) (*n* = 8) 12 m: 0.25 ± 0.10 (0.10–0.36) (*n* = 8)	Lactoferrin concentration	NR	NS
						Lactoferrin intake (overall)		
						↓FFMI (US4SF)	–3.130 (1.040)	0.002 ^b^
						Lactoferrin intake between 2 and 5 m		
						↓ΔWeight 9–12 m	–0. 871 (0.316)	0.015
						↓ΔFFM (US2FS) 9–12 m	–1.052 (0.465)	0.041
						↑ΔFFMI (US4FS) 2–5 m	3.832 (1.546)	0.042
						Lactoferrin intake at 9 m		
						↑ΔWeight 2–19 m	2.154 (0.562)	0.031
						Lactoferrin intake at 12 m		
						↑ΔFM (BIS) 2–12 m	3.529 (0.587)	0.027
						↑ΔFMI (BIS) 2–12 m	8.065 (0.652)	0.007
						↑Δ%FM (BIS) 2–12 m	41.532 (9.350)	0.047
				Lysozyme, g/L 2 m: 0.11 ± 0.11 (0.13–0.46) (*n* = 14) 5 m: 0.1 1 ± 0.05 (0.08–0.29) (*n* = 20) 9 m: 0.14 ± 0.02 (0.10–0.16) (*n* = 19) 12 m: 0.26 ± 0.21 (0.12–0.65) (*n* = 15)	Lysozyme, g/24 h 2–5 m: 0.09 ± 0.07 (0.06–0.34) (*n* = 17) 9 m: 0.06 ± 0.02 (0.04–0.10) (*n* = 8) 12 m: 0.08 ± 0.05 (0.05–0.17) (*n* = 8)	Lysozyme concentration		
						FMI (BIS): ↓2 m; ↑5, 9 m; ↓12 m	NA ^e^	0.026 ^c^
						FM/FFM (US4SF): ↓2 m; ↑5, 9 m; ↓12 m	NA ^e^	0.045 ^c^
						Lysozyme intake (overall)		
						↑FM (US2SF)	1.350 (1.140)	0.037
						↑FM (US4SF)	2.990 (1.250)	0.004 ^b^
						↑%FM (US4SF)	24.90 (11.20)	0.030
						↑FM/FFM (BIS)	8.510 (3.450)	0.018
						↑FMI (US4SF)	8.010 (2.80)	0.004 ^b^
						↑FM/FFM (BIS)	0.546 (0.261)	0.043
						↑ FM/FFM (US4SF)	0.457 (0.203)	0.028
						Lysozyme intake between 2 and 5 m		
						↑ΔWeight 2–5 m	18.226 (6.038)	0.017
						↑ΔWeight 2–9 m	25.390 (10.229)	0.038
						↑ΔBMI 2–5 m	43.300 (9.247)	0.002
						↑ΔFFM (US4SF) 2–9 m	20.817 (7.992)	0.035
						↑ΔFFM (US4SF) 2–12 m	25.039 (10.134)	0.043
						↑ΔFFMI (US4SF) 2–5 m	33.548 (8.580)	0.006
						↑ΔFM (BIS) 2–5 m	12.699 (3.623)	0.010
						↑ΔFMI (BIS) 2–5 m	28.902 (7.779)	0.008
						↑ΔFMI (US2SF) 2–5 m	29.835 (11.250)	0.033
						Lysozyme intake at 9 m	NR	NS
						Lysozyme intake at 12 m		
						↓ΔFFM (US2SF) 5–12 m	–9.658 (2.578)	0.013
						↓ΔFFMI (US2FS) 5–12 m	–44.270 (3.902)	0.0003 ^b^
						↓ΔFM/FFM (BIS)	–2.948 (1.053)	0.049
				sIgA, g/L 2 m: 0.4 6 ± 0.21 (0.08–0.93) (*n* = 14) 5 m: 0.50 ± 0.18 (0.15–0.81) (*n* = 20) 9 m: 0.62 ± 0.21 (0.23–1.08) (*n* = 19) 12 m: 0.71 ± 0.20 (0.34–1.02) (*n* = 15)	sIgA, g/24 h 2–5 m: 0.38 ± 0.18 (0.15–0.80) (*n* = 17) 9 m: 0.28 ± 0.12 (0.10–0.48) (*n* = 8) 12 m: 0.25 ± 0.09 (0.14–0.38) (*n* = 8)	sIgA concentration		
						↓FM (US4SF)	–0.606 (0.298)	0.045
						sIgA intake (overall)		
						↓ FFM (US2SF)	–1.590 (0.602)	0.012
						↓ FFM (US4SF)	–1.640 (0.703)	0.034
						FFMI (BIS): ↑2–5 m; ↓9, 12 m	NA ^e^	0.019 ^c^
						↓FFMI (US4SF)	–3.520 (1.090)	0.008
						↑%FM (US2SF)	9.390 (4.150)	0.046
						↑FM/FFM (US2SF)	0.206 (0.081)	0.021
						sIgA intake between 2 and 5 m		
						↑ΔBMI 2–5 m	3.771 (1.286)	0.019
						↑ΔFFMI (BIS) 2–5 m	1.859 (0.732)	0.035
						↑ΔFM (US2SF) 2–5 m	2.147 (0.617)	0.007
						↑Δ%FM (US2SF) 2–5 m	22.978 (8.156)	0.020
						↑ΔFMI (US2SF) 2–5 m	4.522 (1.461)	0.015
						↑ΔFM/FFM (US2SF) 2–5 m	0.503 (0.167)	0.015
						sIgA intake at 9 m		
						↑ΔHC 5–9 m	2.644 (0.796)	0.021
						sIgA intake at 12 m		
						↑ΔFM (BIS) 2–12 m	4.239 (0.530)	0.015
						↑ΔFM (US4SF) 2–12 m	3.962 (1.146)	0.041
						↑Δ%FM (BIS) 2–12 m	51.094 (5.864)	0.013
						↑ΔFMI (BIS) 2–12 m	9.589 (0.699)	0.005
						↑ΔFMI (US2SF) 2–12 m	9.002 (1. 957)	0.038
						↑ΔFM/FFM (BIS) 2–12 m	0.850 (0.124)	0.021
Gridneva et al., 2019 Australia [41]	LS, 2–12 m, *n* = 20, 2 m (*n* = 15) 5 m (*n* = 20) 9 m (*n* = 19) 12 m (*n* = 18) EBF up to 5 m	SHM pre-/post-feed, morning sampling ^d^ Enzymatic spectrophotometry (lactose); deproteinisation with trichloroacetic acid, dehydration by sulfuric acid (TCH) for calculation of total HMO concentration	5 m: 819 ± 205 (498–1185) (*n* = 17) 9 m: 502 ± 158 (300–775) (*n* = 8) 12 m: 446 ± 200 (255–795) (*n* = 8) 24 h MI: re-/post-feed test-weighing infant BC: anthropometry, BIS, and US skinfolds BC equations	Total HMO, g/L 2 m: 22.3 ± 10.7 (0–35.8) (*n* = 15) 5 m: 16.4 ± 9.9 (2.3–29.9) (*n* = 20) 9 m: 22.5 ± 9.2 (0–36.9) (*n* = 19) 12 m: 21.4 ± 22.3 (3.0–62.2) (*n* = 14)	Total HMO, g/24 h 2–5 m: 12.0 ± 6.0 (2.0–21.6) (*n* = 17) 9 m: 10.8 ± 5.4 (0–15.7) (*n* = 8) 12 m: 12.0 ± 18.5 (1.5–49.5) (*n* = 8)	Total HMO concentration		
						↑Length	0.031 (0.014)	0.036
						↑Weight	0.009 (0.004)	0.038
						BMI: ↑2 m; ↓5, 9, 12 m	NA ^e^	0.027 ^c^
						↑FFM (BIS)	0.015 (0.004)	< 0.001 ^b^
						↑FFMI (BIS)	0.018 (0.007)	0.008 ^b^
						FM (BIS): ↑2 m; ↓5, 9, 12 m	NA ^e^	0.039 ^c^
						%FM (BIS): ↑2 m; ↓5, 9, 12 m	NA ^e^	0.005 ^b,c^
						FMI (BIS): ↑2 m; ↓5, 9, 12 m	NA ^e^	0.003 ^b,c^
						FM/FFM (BIS): ↑2 m; ↓5, 9, 12 m	NA ^e^	0.006 ^b,c^
						Total HMO intake (overall)		
						↑FM (US4SF)	0.020 (0.008)	0.010
						↑%FM (US4 FF)	0.168 (0.074)	0.025
						↓FMI (BIS)	–0.024 (0.017)	0.049
						↑FMI (US4SF)	0.040 (0.018)	0.034
						↓FM/FFM (BIS)	–0.002 (0.001)	0.024
						↑FM/FFM (US4SF)	0.003 (0.001)	0.027
						Total HMO intake between 2 and 5 m		
						↑ΔHC 5–12 m	0.047 (0.018)	0.023
						Total HMO intake at 9 m		
						↓ΔBMI 2–12 m	–0.401 (0.082)	0.040
						↓ΔFFMI (BIS) 5–12 m	–0.110 (0.043)	0.049
						↓ΔFFMI (US4SF) 9–12 m	–0.169 (0.058)	0.032
						↓ΔFMI (BIS) 5–12 m	–0.294 (0.097)	0.029
						Total HMO intake at 12 m		
						↓Δ%FM (BIS) 5–12 m	–0.184 (0.070)	0.047
						↓ΔFMI (BIS) 5–12 m	–0.052 (0.017)	0.032
						↓ΔFM/FFM (BIS) 5–12 m	–0.004 (0.001)	0.021
Gridneva et al., 2018 (b) Australia [43]	LS, 2–12 m, *n* = 20, 2 m (*n* = 13) 5 m (*n* = 20) 9 m (*n* = 18) 12 m (*n* = 13) EBF to 5 m	WHM (adiponectin), SHM and WHM (leptin), pre-/post-feed, morning sampling ^d^ ELISA (adiponectin, whole and skim milk leptin)	5 m: 819 ± 205 (498–1185) (*n* = 17) 9 m: 502 ± 158 (300–775) (*n* = 8) 12 m: 446 ± 200 (255–795) (*n* = 8) 24 h MI: pre-/post-feed test-weighing infant BC: anthropometry, BIS, and US skinfolds BC equations	Adiponectin, ng/mL 2 m: 11.14 ± 5.79 (6.61–21.56) (*n* = 15) 5 m: 8.42 ± 1.69 (6.18–22.58) (*n* = 20) 9 m: 8.44 ± 1.33 (6.41–12.86) (*n* = 18) 12 m: 11.22 ± 4.22 (5.66–19.38) (*n* = 15)	Adiponectin, ng/24 h 2–5 m: 7976 ± 4480 (3771–22439) (*n* = 17) 9 m: 4446 ± 1645 (2142–6673) (*n* = 8) 12 m: 3922 ± 1431 (2511–6352) (*n* = 8)	Adiponectin concentration		
						↓FFM (US4SF)	–0.032 (0.015)	0.025
						Adiponectin intake (overall)		
						HC: ↓2–5 m; ↑9 m; ↓12 m	NA ^e^	0.026 ^c^
						BMI: ↑2–5 m; ↓9, 12 m	NA ^e^	0.016 ^c^
						↓FFM (US4SF)	–0.0001 (0.00002)	0.005 ^b^
						↓FFMI (US4SF)	–0.0001 (0.00004)	0.009 ^b^
						↑FM (US4SF)	0.004 (0.001)	<0.001 ^b^
						↑%FM (US4SF)	0.0007 (0.0002)	<0.001 ^b^
						↑FMI (US2SF)	0.0001 (0.00004)	0.039
						↑FMI (US4SF)	0.0001 (0.00004)	<0.001 ^b^
						Adiponectin intake between 2 and 5 m		
						↑ΔLength 5–9 m	0.0002 (0.0001)	0.010
						↑ΔFFM (US4SF) 2–9 m	0.0001 (0.00002)	0.036
						↑ΔFFM (US4SF) 2–12 m	0.0001 (0.00003)	0.043
						↓ΔFFM (US4SF) 5–9 m	–0.0001 (0.00002)	0.011
						↑ΔFFM (US4SF) 5–12 m	0.0001 (0.00003)	0.018
						↑ΔFFMI (BIS) 2–12 m	0.0001 (0.00004)	0.029
						↑ΔFFMI (US2SF) 2–12 m	0.0001 (0.00004)	0.026
						↑ΔFFMI (US4SF) 2–12 m	0.0002 (0.00004)	0.009
						↓ΔFM (US4SF) 5–12 m	–0.0001 (0.00002)	0.049
						↓Δ%FM (US4SF) 2–9 m	–0.001 (0.0002)	0.044
						↓Δ%FM (US4SF) 5–9 m	–0.001 (0.0003)	0.044
						↓Δ%FM (US4SF) 5–12 m	–0.001 (0.0002)	0.007
						↓Δ%FM (US4SF) 9–12 m	–0.0001 (0.0002)	0.047
						Adiponectin intake at 9 m		
						↓ΔHC 9–12 m	–0.0003 (0.0001)	0.017
						Adiponectin intake at 12 m		
						↓ΔFFMI (US4SF) 5–12 m	–0.001 (0.0002)	0.020
						↑ΔFM (BIS) 2–12 m	0.001 (0.0001)	0.018
						↑ΔFM (US2SF) 2–12 m	0.001 (0.0001)	0.012
						↑Δ%FM (BIS) 2–12 m	0.007 (0.001)	0.011
						↑Δ%FM (US2SF) 2–12 m	0.009 (0.002)	0.013
						↑ΔFMI (US2SF) 2–12 m	0.001 (0.0001)	0.005
				Whole milk leptin, ng/mL 2 m: 0.50 ± 0.18 (0.24–0.77) (*n* = 15) 5 m: 0.49 ± 0.17 (0.23–0.71) (*n* = 20) 9 m: 0.56 ± 0.11 (0.42–0.67) (*n* = 18) 12 m: 0.50 ± 0.11 (0.34–0.74) (*n* = 15)	Whole milk leptin, ng/24 h 2–5 m: 362 ± 173 (162–841) (*n* = 17) 9 m: 280 ± 73 (132–349) (*n* = 8) 12 m: 219 ± 90 (122–350) (*n* = 8)	Whole milk leptin concentration		
						FFM (BIS): ↓2, 5 m; ↑9, 12 m	NA ^e^	0.016 ^c^
						Whole milk leptin intake (overall)		
						FFMI (US4SF): ↑2–5 m; ↓9, 12 m	NA ^e^	0.036 ^c^
						Whole milk leptin intake between 2 and 5 m		
						↑ΔLength 2–9 m	0.008 (0.003)	0.022
						Whole milk leptin intake at 9 m		
						↑ΔLength 2–12 m	0.041 (0.004)	0.007
						Whole milk leptin intake at 12 m		
						↓ΔBMI 9–12 m	–0.008 (0.006)	0.019
						↓ΔFFMI (US2SF) 5–12 m	–0.012 (0.004)	0.040
						↓ΔFFMI (US4SF) 5–12 m	–0.012 (0.002)	0.007
						↑ΔFM (BIS) 2–12 m	0.005 (0.001)	0.046
						↑ΔFM (US2SF) 2–12 m	0.005 (0.0003)	0.0006 ^b^
						↑Δ%FM (BIS) 2–12 m	0.065 (0.015)	0.049
						↑Δ%FM (US2SF) 2–12 m	0.073 (0.004)	0.0004 ^b^
						↑ΔFMI (US2SF) 2–12 m	0.012 (0.002)	0.018
				Skim milk leptin, ng/mL 2 m: 0.34 ± 0.20 (0.20–0.84) (*n* = 15) 5 m: 0.26 ± 0.08 (0.20–0.40) (*n* = 20) 9 m: 0.21 ± 0.02 (0.19–0.27) (*n* = 18) 12 m: 0.21 ± 0.03 (0.19–0.40) (*n* = 15)	Skim milk leptin, ng/24 h 2–5 m: 200 ± 81 (106–402) (*n* = 17) 9 m: 114 ± 38 (62–172) (*n* = 8) 12 m: 93 ± 36 (51–159) (*n* = 8)	Skim milk leptin concentration		
						↓ΔHC	–1.85 (0.84)	0.028
						Skim milk leptin intake (overall)		
						BMI: ↑2–5 m; ↓9, 12 m	NA ^e^	0.004 ^b,c^
						FFMI (US4SF): ↓2–5, 9, 12 m	NA ^e^	0.012 ^b,c^
						↑FM (BIS)	0.003 (0.001)	0.025
						↑FM (US2SF): ↑2–5 m; ↓9 m; ↑12 m	NA ^e^	0.007 ^b,c^
						↑FM (US4SF)	0.004 (0.001)	<0.001 ^b^
						↑%FM (US2SF)	0.029 (0.009)	0.001 ^b^
						↑%FM (US4SF)	0.031 (0.009)	0.002 ^b^
						↑FMI (BIS)	0.005 (0.003)	0.038
						↑FMI (US2SF)	0.008 (0.002)	<0.001 ^b^
						↑FMI (US4SF)	0.008 (0.002)	<0.001 ^b^
						Skim milk leptin intake between 2 and 5 m		
						↑ΔWeight 2–9 m	0.005 (0.002)	0.015
						↑ΔWeight 2–12 m	0.005 (0.002)	0.026
						↑ΔBMI 2–5 m	0.006 (0.002)	0.021
						↑ΔFFM (BIS) 2–12 m	0.005 (0.001)	0.013
						↑ΔFFM (US2SF) 2–9 m	0.003 (0.001)	0.012
						↑ΔFFM (US4SF) 2–9 m	0.004 (0.001)	0.010
						↑ΔFFM (US4SF) 2–12 m	0.005 (0.002)	0.018
						↑ΔFM (BIS) 2–5 m	0.002 (0.001)	0.044
						↑ΔFM (US2SF) 2–5 m	0.003 (0.001)	0.029
						↓Δ%FM (US4SF) 5–12 m	–0.027 (0.012)	0.036
						↑ΔFMI (US2SF) 2–5 m	0.007 (0.003)	0.047
						↓ΔFMI (US4SF) 5–12 m	–0.007 (0.003)	0.026
						Skim milk leptin intake at 9 m		
						↑ΔWeight 2–9 m	0.026 (0.004)	0.007
						↑ΔWeight 2–12 m	0.033 (0.008)	0.025
						↑ΔBMI 2–12 m	0.10 (0.02)	0.035
						↑ΔFM (BIS) 2–9 m	0.052 (0.012)	0.046
						Skim milk leptin intake at 12 m		
						↓ΔFFMI (US2SF) 5–12 m	–0.031 (0.007)	0.005
						↓ΔFFMI (US4SF) 5–12 m	–0.025 (0.002)	0.0004 ^b^
						↑ΔFM (US2SF) 2–12 m	0.010 (0.002)	0.022
						↑Δ%FM (US2SF) 2–12 m	0.135 (0.041)	0.046
Kon et al., 2014 Russian Federation [44]	CS, 1, 2, or 3 m, *n* = 103, 1 m (*n* = 32) 2 m (*n* = 34) 3 m (*n* = 33) LWG (<500 g/m, *n* = 18) NWG (500–1000 g/m, *n* = 40) HWG (>1000 g/m, *n* = 45) EBF	SHM, midstream morning sampling ELISA	Combined mean (calculated): 1 m: 736 (*n* = 32) 2 m: 826 (*n* = 34; no LWG) 3 m: 891 (*n* = 33) LWG 1 m: 555 ± 32 (*n* = 9) 2 m: NA 3 m: 753 ± 53 (*n* = 5) NWG 1 m: 849 ± 96 (*n* = 11) 2 m: 752 ± 93 (*n* = 14) 3 m: 896 ± 42 (*n* = 15) HWG 1 m: 768 ± 41 (*n* = 12) 2 m: 878 ± 37 (*n* = 20) 3 m: 937 ± 70 (*n* = 13) 24 h MI: pre-/post-feed test-weighing infant BC: weight gain	Adiponectin, μg/mL LWG 1 m: 1.06 ± 0.10 (*n* = 9) 2 m: NA (*n* = 4, excluded) 3 m: 1.09 ± 0.15 (*n* = 5) NWG 1 m: 1.14 ± 0.09 (*n* = 11) 2 m: 1.04 ± 0.0 (*n* = 14) 3 m: 1.14 ± 0.08 (*n* = 15) HWG 1 m: 1.10 ± 0.09 (*n* = 12) 2 m: 1.15 ± 0.08 (*n* = 20) 3 m: 1.12 ± 0.10 (*n* = 13) Ghrelin, ng/mL LWG 1 m: 0.77 ± 0.22 (*n* = 9) 2 m: NA (*n* = 4, excluded) 3 m: 8.24 ± 4.76 (*n* = 5) NWG 1 m: 7.52 ± 2.63 (*n* = 11) 2 m: 5.06 ± 2.49 (*n* = 14) 3 m: 0.71 ± 0.19 (*n* = 15) HWG 1 m: 2.32 ± 1.19 (*n* = 12) 2 m: 6.52 ± 1.87 (*n* = 20) 3 m: 3.05 ± 1.9 (*n* = 13) IGF-1, ng/mL LWG 1 m: 3.95 ± 1.86 (*n* = 9) 2 m: NA (*n* = 4, excluded) 3 m: 3.15 ± 1.22 (*n* = 5) NWG 1 m: 3.07 ± 1.55 (*n* = 11) 2 m: 3.21 ± 0.74 (*n* = 14) 3 m: 7.13 ± 1.29 (*n* = 15) HWG 1 m: 7.92 ± 3.72 (*n* = 12) 2 m: 6.97 ± 2.05 (*n* = 20) 3 m: 12.20 ± 2.41 (*n* = 13) Skim milk leptin, ng/mL LWG 1 m: 1.63 ± 0.27 (*n* = 9) 2 m: NA (*n* = 4, excluded) 3 m: 1.35 ± 0.31 (*n* = 5) NWG 1 m: 1.55 ± 0.17 (*n* = 11) 2 m: 1.83 ± 0.23 (*n* = 14) 3 m: 3.29 ± 0.70 (*n* = 15) HWG 1 m: 1.53 ± 0.29 (*n* = 12) 2 m: 2.20 ± 0.28 (*n* = 20) 3 m: 3.57 ± 1.37 (*n* = 13)	Adiponectin, μg/24 h LWG 1 m: 569 ± 56 (*n* = 9) 2 m: NA (*n* = 4, excluded) 3 m: 852 ± 130 (*n* = 5) NWG 1 m: 918 ± 202 (*n* = 11) 2 m: 736 ± 130 (*n* = 14) 3 m: 898 ± 133 (*n* = 15) HWG 1 m: 810 ± 59 (*n* = 12) 2 m: 1030 ± 82 (*n* = 20) 3 m: 1051 ± 116 (*n* = 13) Ghrelin, ng/24 h LWG 1 m: 428 ± 246 (*n* = 9) 2 m: NA (*n* = 4, excluded) 3 m: 6288 ± 3855 (*n* = 5) NWG 1 m: 845 ± 445 (*n* = 11) 2 m: 5343 ± 3938 (*n* = 14) 3 m: 345 ± 112 (*n* = 15) HWG 1 m: 715 ± 279 (*n* = 12) 2 m: 2304 ± 1197 (*n* = 20) 3 m: 488 ± 234 (*n* = 13) IGF-1, ng/24 h LWG 1 m: 1789 ± 1175 (*n* = 9) 2 m: NA (*n* = 4, excluded) 3 m: 2252 ± 822 (*n* = 5) NWG 1 m: 2894 ± 1493 (*n* = 11) 2 m: 2898 ± 804 (*n* = 14) 3 m: 6289 ± 1059 (*n* = 15) HWG 1 m: 5653 ± 2666 (*n* = 12) 2 m: 5690 ± 1567 (*n* = 20) 3 m: 11351 ± 2990 (*n* = 13) Skim milk leptin, ng/24 h LWG 1 m: 860 ± 225 (*n* = 9) 2 m: NA (*n* = 4, excluded) 3 m: 1096 ± 150 (*n* = 5) NWG 1 m: 1291 ± 619 (*n* = 11) 2 m: 1237 ± 272 (*n* = 14) 3 m: 3463 ± 1232 (*n* = 15) HWG 1 m: 1584 ± 420 (*n* = 12) 2 m: 2777 ± 567 (*n* = 20) 3 m: 4979 ± 1959 (*n* = 13)	Adiponectin concentration		
						No significant difference between LWG, NWG, and HWG groups at any time point	Concentrations by group and time point are on the left	NS
						Adiponectin intake		
						↑Adiponectin intake at 1 m in the HWG group compared with the LWG group	Intakes by group and time point are on the left	<0.05
		
		
		
		
		
		
		
		
		
		
						Ghrelin concentration		
						↑Ghrelin concentration at 1 m in the HWG group compared with the NWG and LWG groups		<0.05
						Ghrelin intake		
						No significant difference between the LWG, NWG, and HWG groups at any time point		NS
		
		
		
		
		
		
		
		
		
		
		
		
						IGF-1 concentration		
						↑IGF-1 concentration at 3 m in the HWG group compared with the LWG group		<0.05
						IGF-1 intake		
						↑IGF-1 intake at 3 m in the HWG group compared with the LWG group		<0.05
						↑IGF-1 intake at 3 m in the NWG group compared with the LWG group		<0.05
		
		
		
		
		
		
		
		
		
						Skim milk leptin concentration		
						↑Skim milk leptin concentration at 3 m in NWG compared with LWG group		<0.05
						Skim milk leptin intake		
						↑Skim milk leptin intake at 2 m in HWG compared with NWG group		<0.05
		
		
		
		
		
		
		
		
		
		
		
		

^a^ Data are mean ± SD, median [IQR], mean difference (95% CI), and/or β (parameter estimate) (SEE); only results significant prior to and post multiple comparison adjustment are presented in detail. ^b^ The results are significant after multiple comparisons. ^c^ *p*-value reported is for significant interaction. ^d^ Sampling time reported by the author on request. ^e^ Overall ß (SE) are not available when significant interaction with age is present; individual ß (SE) reported for 2, 5, 9, and 12 months. BC, body composition; BIS, bioelectrical impedance spectroscopy; BMI, body mass index; CS, cross-sectional study; EBF, exclusively breastfed; FFM, fat-free mass; FFMI, fat–free mass index; FM, fat mass; FMI, fat mass index; HC, head circumference; HWG, high weight gain; IGF-1, insulin-like growth factor–1; LBM, lean body mass; LAZ, length-for-age z score; LS, longitudinal study; LWG, low weight gain; m, month; MI, milk intake; NA, not available; NMR, ^1^ H-nuclear magnetic resonance; NR, not reported; NS, not significant; NW, normal weight; NWG, normal weight gain; OW, overweight; SDS, standard deviation scores; SEE, standard error of estimate; SHM, skim human milk; sIgA, secretory immunoglobulin A; US, ultrasound; US2SF, ultrasound 2-skinfolds; US4SF, ultrasound 4-skinfolds; w, week; WAZ, weight-for-age z score; WLZ, weight-for-length z score; WHM, whole human milk. ↓, lower; ↑, higher.

**Table A4:** Summary of intakes of human milk bioactive molecules presented by time postpartum.

Human Milk Bioactive Molecules	Time Postpartum	Sample Size	Human Milk Sample Type	24 h Intake of Bioactive Molecules	Comments	Author, Year
Adiponectin	1 m	*n* = 9 *n* = 11 *n* = 12	Skim milk	569 ± 56 μg/24 h ^a^ 918 ± 202 810 ± 59	LWG NWG HWG	Kon et al., 2014 [44]
	2 m	*n* = 4 *n* = 14 *n* = 20	Skim milk	NA 736 ± 130 μg/24 h 1030 ± 82	LWG NWG HWG	Kon et al., 2014 [44]
	3 m	*n* = 5 *n* = 15 *n* = 13	Skim milk	852 ± 130 μg/24 h 898 ± 133 1051 ± 116	LWG NWG HWG	Kon et al., 2014 [44]
	2–5 m	*n* = 17	Whole milk	7976 ± 4480 (3771–22,439) ng/24 h		Gridneva et al., 2018 (b) [43]
	9 m	*n* = 8	Whole milk	4446 ± 1645 (2142–6673) ng/24 h		Gridneva et al., 2018 (b) [43]
	12 m	*n* = 8	Whole milk	3922 ± 1431 (2511–6352) ng/24 h		Gridneva et al., 2018 (b) [43]
Whole milk leptin	2–5 m	*n* = 17	Whole milk	362 ± 173 (162–841) ng/24 h		Gridneva et al., 2018 (b) [43]
	9 m	*n* = 8	Whole milk	280 ± 73 (132–349) ng/24 h		Gridneva et al., 2018 (b) [43]
	12 m	*n* = 8	Whole milk	219 ± 90 (122–350) ng/24 h		Gridneva et al., 2018 (b) [43]
Skim milk leptin	1 m	*n* = 9 *n* = 11 *n* = 12	Skim milk	860 ± 225 ng/24 h 1291 ± 619 1584 ± 420	LWG NWG HWG	Kon et al., 2014 [44]
	2 m	*n* = 4 *n* = 14 *n* = 20	Skim milk	NA 1237 ± 272 ng/24 h 2777 ± 567	LWG NWG HWG	Kon et al., 2014 [44]
	3 m	*n* = 5 *n* = 15 *n* = 13	Skim milk	1096 ± 150 ng/24 h 3463 ± 1232 4979 ± 1959	LWG NWG HWG	Kon et al., 2014 [44]
	2–5 m	*n* = 17	Skim milk	200 ± 81 (106–402) ng/24 h		Gridneva et al., 2018 (b) [43]
	9 m	*n* = 8	Skim milk	114 ± 38 (62–172) ng/24 h		Gridneva et al., 2018 (b) [43]
	12 m	*n* = 8	Skim milk	93 ± 36 (51–159) ng/24 h		Gridneva et al., 2018 (b) [43]
Insulin	3 m	*n* = 45	Whole milk	1.16 ± 0.78 (0.30–4.17) ng/24 h		Cheema et al., 2021 [37]
Ghrelin	1 m	*n* = 9 *n* = 11 *n* = 12	Skim milk	428 ± 246 ng/24 h 845 ± 445 715 ± 279	LWG NWG HWG	Kon et al., 2014 [44]
	2 m	*n* = 4 *n* = 14 *n* = 20	Skim milk	NA 5343 ± 3938 ng/24 h 2304 ± 1197	LWG NWG HWG	Kon et al., 2014 [44]
	3 m	*n* = 5 *n* = 15 *n* = 13	Skim milk	6288 ± 3855 ng/24 h 345 ± 112 488 ± 234	LWG NWG HWG	Kon et al., 2014 [44]
IGF-1	1 m	*n* = 9 *n* = 11 *n* = 12	Skim milk	1789 ± 1175 ng/24 h 2894 ± 1493 5653 ± 2666	LWG NWG HWG	Kon et al., 2014 [44]
	2 m	*n* = 4 *n* = 14 *n* = 20	Skim milk	NA 2898 ± 804 ng/24 h 5690 ± 1567	LWG NWG HWG	Kon et al., 2014 [44]
	3 m	*n* = 5 *n* = 15 *n* = 13	Skim milk	2252 ± 822 ng/24 h 6289 ± 1059 11,351 ± 2990	LWG NWG HWG	Kon et al., 2014 [44]
Lactoferrin	2–5 m	*n* = 17	Skim milk	0.29 ± 0.12 (0.06–0.51) g/24 h		Gridneva et al., 2020 [40]
	9 m	*n* = 8	Skim milk	0.31 ± 0.21 (0.13–0.74) g/24 h		Gridneva et al., 2020 [40]
	12 m	*n* = 8	Skim milk	0.25 ± 0.10 (0.10–0.36) g/24 h		Gridneva et al., 2020 [40]
Lysozyme	2–5 m	*n* = 17	Skim milk	0.09 ± 0.07 (0.06–0.34) g/24 h		Gridneva et al., 2020 [40]
	9 m	*n* = 8	Skim milk	0.06 ± 0.02 (0.04–0.10) g/24 h		Gridneva et al., 2020 [40]
	12 m	*n* = 8	Skim milk	0.08 ± 0.05 (0.05–0.17) g/24 h		Gridneva et al., 2020 [40]
sIgA	2–5 m	*n* = 17	Skim milk	0.38 ± 0.18 (0.15–0.80) g/24 h		Gridneva et al., 2020 [40]
	9 m	*n* = 8	Skim milk	0.28 ± 0.12 (0.10–0.48) g/24 h		Gridneva et al., 2020 [40]
	12 m	*n* = 8	Skim milk	0.25 ± 0.09 (0.14–0.38) g/24 h		Gridneva et al., 2020 [40]
HMOs	3 m	*n* = 47	Whole milk	See paper for intakes of 19 individual HMOs [36]	In entire cohort and by maternal secretor status	Cheema et al., 2022 [36]
Total HMOs	2–5 m	*n* = 17	Skim milk	12.0 ± 6.0 (2.0–21.6) g/24 h		Gridneva et al., 2019 [41]
	9 m	*n* = 8	Skim milk	10.8 ± 5.4 (0–15.7) g/24 h		Gridneva et al., 2019 [41]
	12 m	*n* = 8	Skim milk	12.0 ± 18.5 (1.5–49.5) g/24 h		Gridneva et al., 2019 [41]

^a^ Data are mean ± SD (standard deviation) and min–max where available. HMO, human milk oligosaccharides; HWG, high weight gain; LWG, low weight gain; m, month; MFGM, milk fat globule membrane; NA, not available; NWG, normal weight gain; sIgA, secretory immunoglobulin A; w, week.
