# Supplementary material for: Relationships between the Intakes of Human Milk Components and Body Composition of Breastfed Infants: A Systematic Review
Source: Nutrients. 2023 May 18;15(10):2370. doi: 10.3390/nu15102370 (PMC10223764; doi:10.3390/nu15102370)
Supplement: Supplementary file 1 [file nutrients-15-02370-s001.zip › nutrients-2383551-supplementary.pdf]

*Review*

# Relationships between the Intakes of Human Milk Components and Body Composition of Breastfed Infants: A Systematic Review

**Isabella Norrish [1], Azhar Sindi [2,3], Vanessa S. Sakalidis [1], Ching Tat Lai [1], Jacki L. McEachran [1], Mya Thway Tint [4,5], Sharon L. Perrella [1], Mark P. Nicol [6], Zoya Gridneva [1,*,†] and Donna T. Geddes [1,†]**

## Supplementary Material: Search Strategy for infant intake of human milk components and infant body composition and growth in MEDLINE, EMBASE, and CINAHL PLUS

**Table S1.** Ovid MEDLINE(R) ALL <1946 to May 17, 2022>

| | |
|---|---|
| 1 | *Milk, Human/ |
| 2 | (breast milk or human milk or milk, breast or milk, human).mp. |
| 3 | 1 or 2 |
| 4 | (human milk component* or human milk composition or protein or carbohydrate* or fat or macronutrient* or bioactive component* or hormone* or adipokine* or cytokine* or leptin or ghrelin or adiponectin or IGF-1 orcasein or whey or total protein or lactose or human milk oligosaccharide* or HMOs or lactoferrin or lysozyme or secretory immunoglobulin A or immunomodulatory or appetite hormone*).ti,ab. |
| 5 | 3 and 4 |
| 6 | *Body Composition/ge, im, ph [Genetics, Immunology, Physiology] |
| 7 | (body composition or body compositions or composition, body or compositions, body).mp |
| 8 | *Growth/ge, im, ph [Genetics, Immunology, Physiology] |
| 9 | (infant adj3 (growth or bmi or body mass index or weight gain or body weight*)).mp |
| 10 | 6 or 7 or 8 or 9 |
| 11 | 5 and 10 |
| 12 | limit 11 to (english language and humans) |

[mp=title, abstract, heading word, drug trade name, original title, device manufacturer, drug manufacturer, device trade name, keyword, floating subheading word]
[ti,ab=title, abstract]

**Table S2.** EMBASE <1974 to 2022 May 18>

| | |
|---|---|
| 1 | *Milk, Human/ |
| 2 | (breast milk or human milk or milk, breast or milk, human).mp. |
| 3 | 1 or 2 |
| 4 | human milk component* or human milk composition or protein or carbohydrate* or fat or macronutrient* or bioactive component* or hormone* or adipokine* or cytokine* or leptin or ghrelin or adiponectin or IGF-1 orcasein or whey or total protein or lactose or human milk oligosaccharide* or HMOs or lactoferrin or lysozyme or secretory immunoglobulin A or immunomodulatory or appetite hormone*.ti,ab |
| 5 | 3 and 4 |
| 6 | *body composition/ |
| 7 | (body composition or body compositions or composition, body or compositions,body).mp. |
| 8 | *growth/ |
| 9 | (infant adj3 (growth or bmi or body mass index or weight gain or body weight*)).mp. |
| 10 | 6 or 7 or 8 or 9 |
| 11 | 5 and 10 |
| 12 | limit 11 to (human and english language) |

[mp=title, abstract, heading word, drug trade name, original title, device manufacturer, drug manufacturer, device trade name, keyword, floating subheading word]
[ti,ab=title, abstract]

**Table S3**. CINAHL PLUS < 1937 to 2022 May 18>

| | |
|---|---|
| 1 | MH "Milk, Human+ |
| 2 | breast milk or human milk or milk, breast or milk, human |
| 3 | 1 or 2 |
| 4 | human milk component* or human milk composition or protein or carbohydrate* or fat or macronutrient* or bioactive component* or hormone* or adipokine* or cytokine* or leptin or ghrelin or adiponectin or IGF-1 orcasein or whey or total protein or lactose or human milk oligosaccharide* or HMOs or lactoferrin or lysozyme or secretory immunoglobulin A or immunomodulatory or appetite hormone* |
| 5 | 3 and 4 |
| 6 | MM "Body Composition" |
| 7 | body composition or body compositions or composition, body or compositions, body |
| 8 | MM "Growth" |
| 9 | infant adj3 (growth or bmi or body mass index or weight gain or body weight*) |
| 10 | 6 or 7 or 8 or 9 |
| 11 | 5 and 10 |
| 12 | limit 11 to (human and english language) |

MH- Exact Subject Heading
MM- Exact Major Subject Heading
[Expanders - Apply related words; Apply equivalent subjects]

**Table S4.**

| | | | |
|---|---|---|---|
| S60 | S59 | Limiters - English Language; Human | 134 |
| S59 | S53 AND S58 | | 195 |
| S58 | S54 OR S55 OR S56 OR S57 | Expanders - Apply related words; Apply equivalent subjects Search modes - Boolean/Phrase | 28,377 |
| S57 | infant adj3 (growth or bmi or body mass index or weight gain or body weight*) | Expanders - Apply related words; Apply equivalent subjects Search modes - Boolean/Phrase | 312 |
| S56 | MM "Growth" | Expanders - Apply related words; Apply equivalent subjects Search modes - Boolean/Phrase | 3,258 |
| S55 | body composition or body compositions or composition, body or compositions, body | Expanders - Apply related words; Apply equivalent subjects Search modes - Boolean/Phrase | 25,312 |
| S54 | MM "Body Composition" | Expanders - Apply related words; Apply equivalent subjects Search modes - Boolean/Phrase | 8,112 |
| S53 | S51 AND S52 | Expanders - Apply related words; Apply equivalent subjects Search modes - Boolean/Phrase | 2,661 |
| S52 | human milk component* or human milk composition or protein or carbohydrate* or fat or macronutrient* or bioactive component* or hormone* or adipokine* or cytokine* or leptin or ghrelin or adiponectin or IGF-1 orcasein or whey or total protein or lactose or human milk oligosaccharide* or HMOs or lactoferrin or lysozyme or secretory immunoglobulin A or immunomodulatory or appetite hormone* | Expanders - Apply related words; Apply equivalent subjects Search modes - Boolean/Phrase | 484,837 |
| S51 | S49 OR S50 | Expanders - Apply related words; Apply equivalent subjects Search modes - Boolean/Phrase | 10,945 |
| S50 | breast milk or human milk or milk, breast or milk, human | Expanders - Apply related words; Apply equivalent subjects Search modes - Boolean/Phrase | 10,915 |
| S49 | MH "Milk, Human+" | Expanders - Apply related words; Apply equivalent subjects Search modes - Boolean/Phrase | 7,368 |
